# Integrin-Associated Focal Adhesion Kinase Protects Human Embryonic Stem Cells from Apoptosis, Detachment, and Differentiation

**DOI:** 10.1016/j.stemcr.2016.07.006

**Published:** 2016-08-09

**Authors:** Loriana Vitillo, Melissa Baxter, Banu Iskender, Paul Whiting, Susan J. Kimber

**Affiliations:** 1 North West Embryonic Stem Cell Centre, Faculty of Life Sciences, University of Manchester, Manchester M13 9PT, UK; 2 Pfizer Neusentis, The Portway Building, Granta Park, Cambridge CB21 6GS, UK

## Abstract

Human embryonic stem cells (hESCs) can be maintained in a fully defined niche on extracellular matrix substrates, to which they attach through integrin receptors. However, the underlying integrin signaling mechanisms, and their contribution to hESC behavior, are largely unknown. Here, we show that focal adhesion kinase (FAK) transduces integrin activation and supports hESC survival, substrate adhesion, and maintenance of the undifferentiated state. After inhibiting FAK kinase activity we show that hESCs undergo cell detachment-dependent apoptosis or differentiation. We also report deactivation of FAK downstream targets, AKT and MDM2, and upregulation of p53, all key players in hESC regulatory networks. Loss of integrin activity or FAK also induces cell aggregation, revealing a role in the cell-cell interactions of hESCs. This study provides insight into the integrin signaling cascade activated in hESCs and reveals in FAK a key player in the maintenance of hESC survival and undifferentiated state.

## Highlights

• Integrin activation in hESCs is transduced by FAK kinase
• FAK protects hESCs from anoikis upstream of the AKT/MDM2/p53 cascade
• FAK is required for maintenance of low contractility and attachment of hESCs
• Prolonged inhibition of FAK induces non-biased hESCs differentiation

## Graphical Abstract

**Figure:**
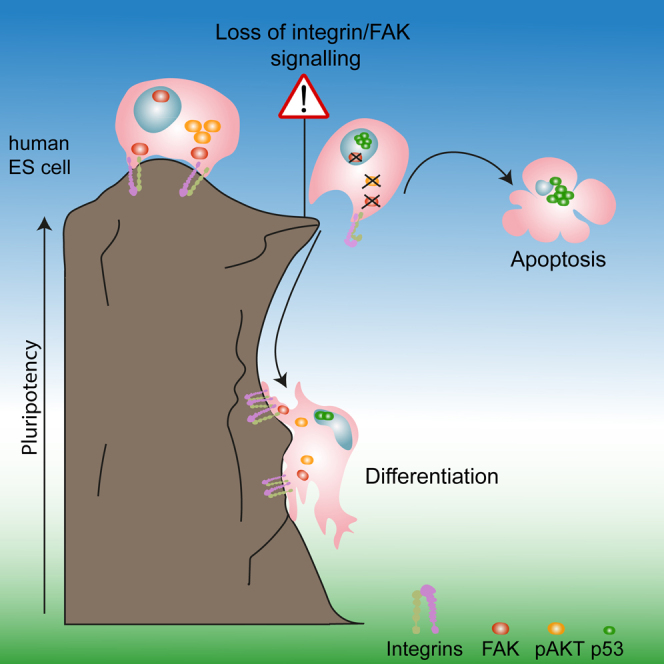

## Teaser

In this research, Kimber and colleagues reveal the critical role of the extracellular matrix-dependent signaling in controlling human embryonic stem cell behavior. Such cues are principally mediated by focal adhesion kinase signaling, which links to intrinsic pluripotent stem cell regulators, such as AKT and p53, preventing detachment, apoptosis, and differentiation.

## Introduction

Human embryonic stem cells (hESCs) are pluripotent stem cells that exhibit epithelial-like features resembling the epiblast epithelium of the post-implantation embryo (Nichols and Smith, 2009). Similarly to epithelial cells, hESCs are dependent on E-cadherin-mediated cell-cell contacts and anchorage to the extracellular matrix (ECM) via integrin receptors (Ohgushi et al., 2010, Braam et al., 2008). Various studies have established the efficacy of integrin engagement with ECM substrates in supporting hESC self-renewal and pluripotency (Braam et al., 2008, Baxter et al., 2009, Miyazaki et al., 2012, Soteriou et al., 2013, Rodin et al., 2014). However, the specific nature and role of downstream signaling from integrins in hESCs remains largely unexplored.

One of the key functions of the ECM in epithelial cells is to prevent a common form of apoptosis, anoikis, or “homelessness” of cells that have lost contact with the matrix (Frisch and Francis, 1994). Anoikis is executed via the mitochondrion and results in activation of caspase downstream of integrin-associated pathways (Gilmore et al., 2000). ECM-integrin interaction initiates signaling, promoting the assembly of cytoplasmic scaffold and kinase proteins at focal adhesions near active integrin clusters (Giancotti and Ruoslahti, 1999). Focal adhesion kinase (FAK), a protein tyrosine kinase, is one of the principal integrin signaling regulators, containing three domains: the protein 4.1, ezrin, radixin, moesin (FERM) domain, the kinase domain, and the focal adhesion targeting domain (Frame et al., 2010). Upon integrin activation FAK localizes at the adhesion site where structural changes displace the inhibitory FERM, allowing autophosphorylation of the Tyr397 (Y397) site, leading to the activation of its intrinsic kinase function and the formation of docking sites for multiple downstream signaling molecules (Frame et al., 2010). Several signaling players directly interact with the Y397 site, e.g., Src, which in turn phosphorylates FAK, promoting further activation, or p130Cas, Grb2, and phosphatidylinositol 3-kinase (PI3K), involved in controlling cytoskeletal rearrangements, cell cycle, and survival (Parsons, 2003). FAK is crucial in preventing anoikis through direct activation of PI3K, via the Y397 site, in turn promoting the pro-survival AKT cascade (Gilmore et al., 2000, Xia et al., 2004). FAK can also leave focal adhesions and act in a kinase-independent manner by localizing in the nucleus where the FERM scaffolds the AKT target MDM2 for ubiquitination of pro-apoptotic p53, leading to its protein degradation (Lim et al., 2008).

Among the repertoire of integrins, the β1-integrin subunit mediates the attachment of hESCs to fibronectin via the α5β1 heterodimer (Baxter et al., 2009), as well as other commonly used ECM (Braam et al., 2008). Although hESCs cultured on ECM have been shown to express active FAK and AKT (Miyazaki et al., 2012, Rodin et al., 2014, Wrighton et al., 2014), the functional contribution of the FAK pathway to hESCs has not been dissected.

Here, we show that integrin activation in hESCs is transduced by FAK to regulate adhesion and prevent the onset of anoikis or differentiation via an AKT/MDM2/p53 cascade. Together, our results reveal a critical role for FAK in the control of hESC fate, as a mediator of integrin signaling crosstalk with key hESC regulatory players.

## Results

### Matrix-Integrin Binding Activates FAK Signaling Upstream of AKT

**Figure 1:**
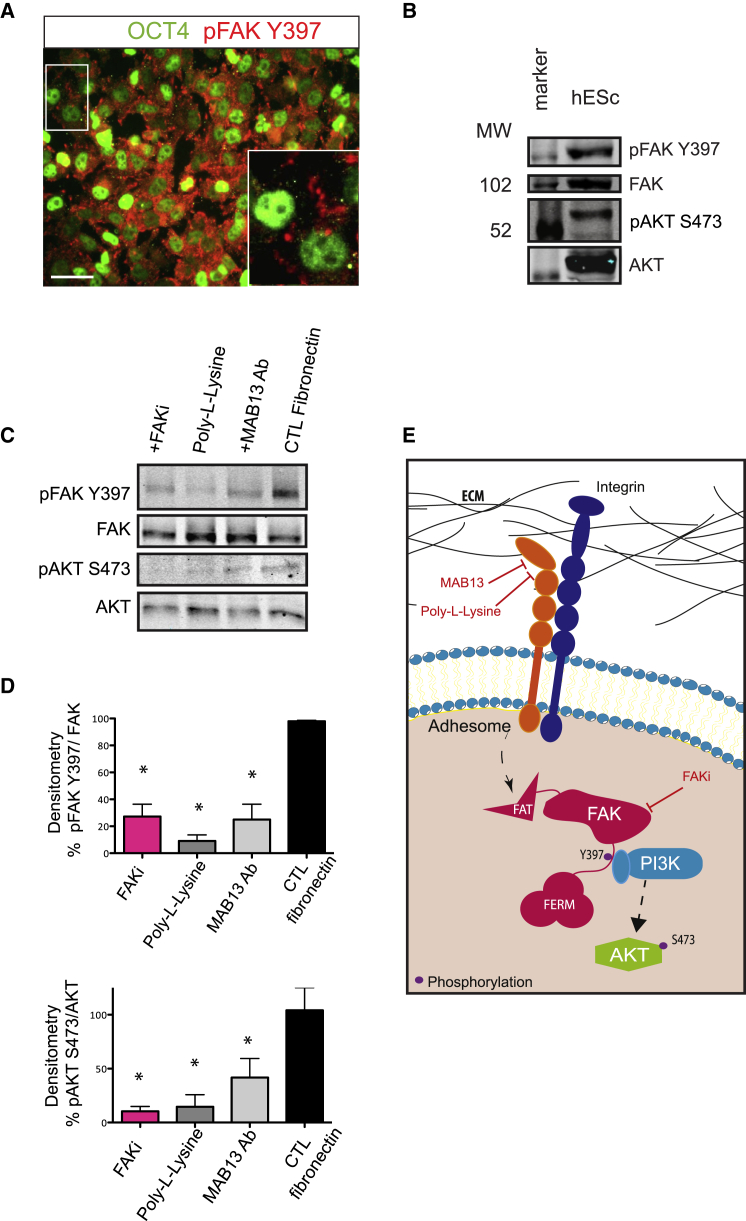
Matrix-Integrin Binding Activates FAK Signaling Upstream of AKT (A) Immunofluorescence of hESCs cultured on fibronectin for 24 hr and stained with antibodies against OCT4 and pFAKY397. Scale bar, 50 μm. (B) Immunoblot of pFAKY397, FAK, pAKTS473, and AKT in hESCs stably cultured on fibronectin. (C) Immunoblot of pFAKY397, FAK, pAKTS473, and AKT in hESCs 1 hr after being plated in the following conditions, on: fibronectin (CTL); fibronectin plus 10 μg/mL of β1-integrin blocking antibody (MAB13); non-integrin activating Poly-L-Lysine substrate or fibronectin plus 2 μM PF562271 (FAKi). (D) Densitometry of immunoblots for pFAKY397/FAK ratio and pAKTS473/AKT ratio for the conditions in (C). Data represent mean + SEM (n = 3 experiments). ^∗^p < 0.05 relative to CTL. (E) Integrin signaling cascade and points of inhibition. See also Figure S1.

To characterize integrin signaling in hESCs cultured on fibronectin, we investigated FAK activation. Immunofluorescence analysis of phosphorylation sites marking FAK activity showed widespread expression of the autophosphorylation Y397 site, induced upon integrin engagement in OCT4-positive cells (Figure 1A). Other phosphorylated residues, created by Src kinase binding to FAKY397 during adhesome assembly, were expressed in a small proportion of cells (Figure S1A) showing that hESCs display active FAK signaling. Importantly, hESCs express high levels of active β1-integrin and the focal adhesion marker paxillin but in a diffuse or punctate distribution, while upon differentiation focal adhesions are visible (Figure S1B). Next, we asked whether FAKY397 is a transducer of fibronectin/β1-integrin binding. hESCs grown on fibronectin had active FAKY397 and its downstream PI3K target AKT Ser473 (S473) (Figure 1B). Conversely, plating hESCs on a non-integrin-activating substrate, Poly-L-Lysine, or blocking β1-integrin selectively with antibody (MAB13), which we previously showed induces hESC detachment from fibronectin (Baxter et al., 2009), reduced FAK and AKT activity (Figures 1C and 1D). We then applied a selective FAK inhibitor, PF562271, which reduced pFAKY397 in a dose-dependent manner (Figures S1C and S1D). Pharmacological inhibition of 80% of FAKY397 with PF562271 at 2 μM (FAKi) was comparable with blocking β1-integrin in reduction of FAK activity (Figures 1C and 1D) without affecting the potential off-target CDK1 (Figure S1E). Moreover, after FAK inhibition there was a reduction of pAKT similar to that seen following integrin inhibition or on Poly-L-Lysine (Figures 1C and 1D). In summary, our data indicate that integrin engagement in hESCs is transduced through FAKY397 and its downstream kinase, AKT (Figure 1E).

### Inhibition of FAK Signaling Induces Cell Blebbing and a Caspase-Dependent Anoikis

**Figure 2:**
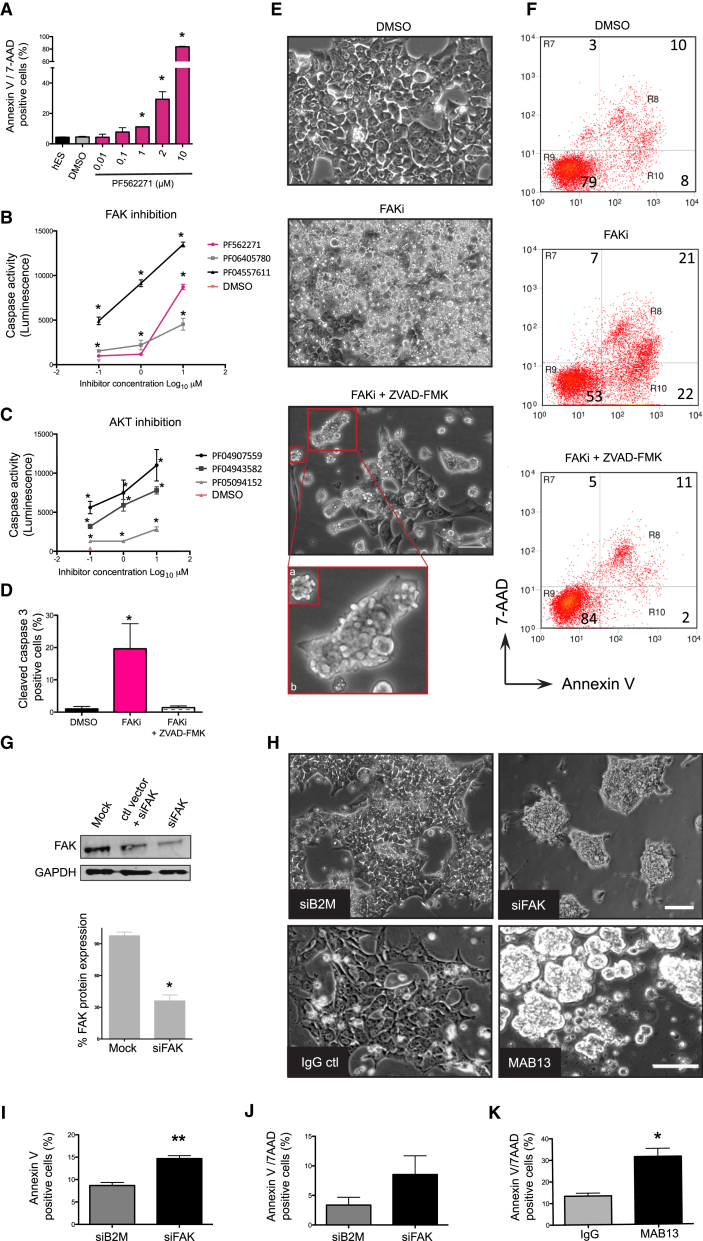
Inhibition of FAK Signaling Induces Cell Blebbing and a Caspase-Dependent Anoikis (A) Quantification of Annexin V/7-AAD positive cells by flow cytometry in hESCs treated for 24 hr with the indicated concentrations of PF562271, DMSO, or untreated. ^∗^p < 0.05 relative to DMSO. (B) Caspase activity in hESCs treated for 5 hr with the indicated concentration of FAK inhibitors. ^∗^p < 0.05 relative to DMSO. (C) Caspase activity in hESCs treated for 5 hr with the indicated concentration of AKT inhibitors. ^∗^p < 0.05 relative to DMSO. (D) Cleaved caspase-3 expression in hESCs treated with DMSO, FAKi only, or with 50 μM ZVAD-FMK for 24 hr. ^∗^p < 0.05 relative to DMSO. (E) Phase images of hESCs treated for 24 hr with DMSO, FAKi only, or with Z-VAD-FMK. Inset shows blebbing in a single cell (a) or groups of cells (b). Scale bar, 100 μm. (F) Dot plots of Annexin V/7-AAD-positive cells in hESCs treated for 24 hr with DMSO, FAKi only, or with Z-VAD-FMK. (G) Immunoblot for FAK and GAPDH in hESCs nucleofected with mock control, control GFP vector (ctl vector) plus FAK siRNA (siFAK), or siFAK for 48 hr. Bottom: protein knockdown efficiency. ^∗^p < 0.05. (H) Top: phase images of hESCs after knockdown with β2-microglobulin siRNA (siB2M) control or siFAK for 66 hr. Bottom: hESCs after treatment with 10 μg/mL of IgG isotype or MAB13 antibody for 24 hr. Scale bars, 100 μm (top) and 50 μm (bottom). (I) Quantification of Annexin V-positive hESCs after knockdown with siB2M or siFAK (66 hr). ^∗∗^p < 0.03. (J) Quantification of Annexin V/7-AAD-positive hESCs after knockdown with siB2M or siFAK (66 hr). (K) Quantification of Annexin V/7-AAD-positive hESCs treated with IgG or MAB13 antibody (24 hr). ^∗^p < 0.05. Data represent mean + SEM (n = 3 experiments). See also Figure S2.

Autophosphorylation of FAK is crucial for the transduction of survival signals by recruiting PI3K that in turn induces the AKT cascade (Xia et al., 2004). Since our data suggested that FAKY397 transduces integrin activation to AKT, we next determined whether FAK kinase activity supports survival of hESCs. hESCs responded to FAK kinase block with PF562271 by detaching from the matrix (Figure 2E) and undergoing apoptosis in a dose-dependent manner (Figure 2A). At the same time, we excluded the possibility that the FAK/Src complex mediated survival, since inhibition of Src did not induce cell detachment and dephosphorylation of AKT even if its target FAKY861 was decreased (Figures S2A and S2B). Similarly, inhibition of the integrin-associated pseudokinase ILK, reported to target AKT in differentiating hESCs (Wrighton et al., 2014), did not affect cell attachment or survival (Figure S2C). Furthermore, inhibition of ILK did not affect pluripotency markers over time (Figure S2D).

To further confirm that the inhibition of FAK kinase is responsible for apoptosis of hESCs, we tested in parallel two other selective FAK inhibitors in both hESCs and human induced pluripotent stem cells (hiPSCs) with a high-throughput assay that measures early caspase activation. All tested FAK inhibitors induced caspase activity in proportion to dose in both hESCs (Figure 2B) and hiPSCs (Figure S2E). In addition, selective inhibitors of AKT induced caspase activity in a similar manner (Figure 2C), supporting AKT as an effector of FAK. Finally, by measuring together caspase activity, viability, and cytotoxicity after FAKi, we found that caspase activity was induced without non-specific cytotoxicity (Figure S2F).

To validate whether FAK-dependent apoptosis relies on caspase activity, we applied the caspase inhibitor Z-VAD-FMK to hESCs treated with FAKi. Immunostaining confirmed that FAKi induced cleaved caspase-3 expression that was inhibited by Z-VAD-FMK (Figure 2D). Floating dead cells, normally present after FAKi, were abolished by Z-VAD-FMK but instead, attached single and groups of hESCs, with prominently blebbing membranes, were observed (Figure 2E). The effects of FAKi on survival, cell blebbing, and caspase activation were confirmed on vitronectin (Figures S2G and S2H).

Cell blebbing is an indicator of cytoskeletal contraction, commonly a result of caspase-3 cleavage of ROCK during apoptosis, leading to increased contractility (Coleman et al., 2001). Our data indicate that FAK inhibition induced cell blebbing independently of caspase, a unique mechanism reported in hESCs after cell-cell dissociation (Ohgushi et al., 2010), but also observed by us in groups of cells. However, caspase activity is required to complete detachment from the ECM, a key feature of anoikis. Moreover, Z-VAD-FMK also rescued FAKi-dependent early (Annexin V) and late (Annexin V/7-AAD) apoptosis, increasing the proportion of live cells, suggesting a suppression of hESC caspase-dependent turnover (Figure 2F). Overall, these results show that FAK kinase activity is required to suppress a caspase-dependent anoikis in hESCs.

### Loss of Total FAK Disrupts hESC Attachment and Leads to Cell Aggregation

Since FAK is a modular protein that functions as a scaffold during adhesome assembly linking integrins to the actin cytoskeleton, we investigated the effect of substantially reducing total FAK on hESCs. Knocking down FAK protein by 70% using small interfering RNA (siFAK) (Figure 2G) induced visible changes in morphology and decreased hESC attachment to fibronectin. Cells formed aggregates similar to those seen after blocking β1-integrin with antibody (Figure 2H), showing that integrin/FAK is required for the transmission of attachment cues in hESCs. Moreover, cell aggregates were also produced in hESCs grown on vitronectin, after blocking its receptor αVβ5-integrin with antibody (Figure S2I). Similarly to the effect of MAB13, pFAK activity was downregulated after inhibition of vitronectin-integrin binding (Figure S2J), suggesting that a common downstream FAK signaling may be activated. Furthermore, apoptosis was increased by both MAB13 and siFAK (Figures 2K and 2I), although late apoptotic cells are fewer in the latter (Figure 2J), strongly supporting the role of β1-integrin and FAK in survival of hESCs. In conclusion, loss of β1-integrin activity or FAK produces a shift from matrix-cell adhesion to cell-cell adhesion with lowered survival, highlighting the role of integrin/FAK signaling in the maintenance of pro-survival adhesion cues in hESCs.

### FAK Localizes in the Nucleus of hESCs and Regulates MDM2/p53 Levels during Anoikis

**Figure 3:**
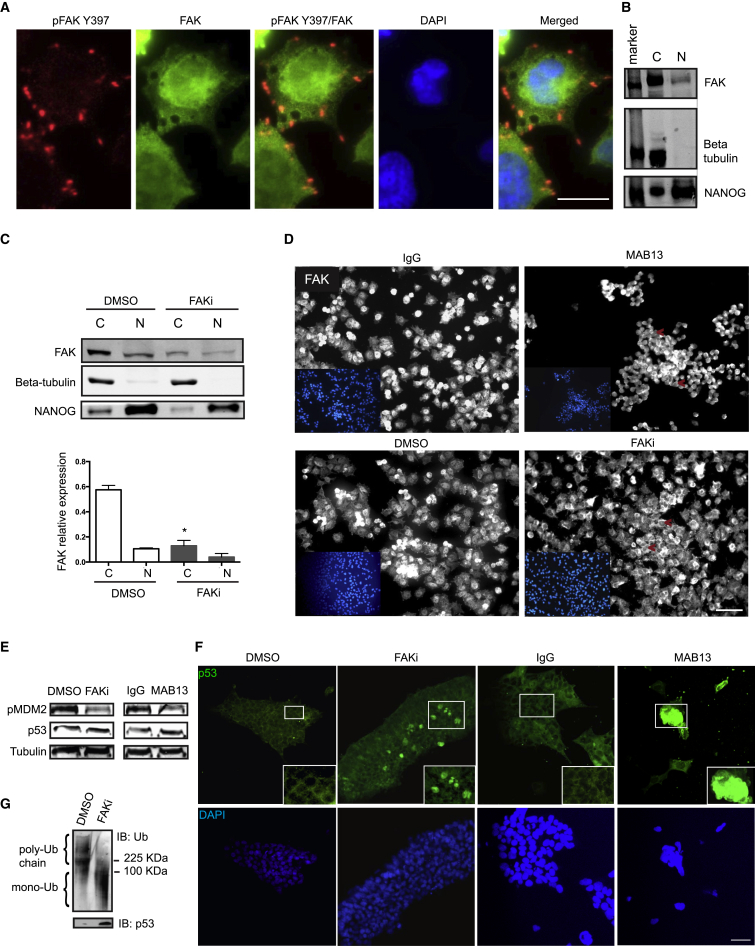
FAK Localizes in the Nucleus of hESCs and Regulates MDM2/p53 Levels during Anoikis (A) Wide-field fluorescence microscopy images of hESCs co-stained with DAPI and antibodies against pFAKY397 and total FAK. Scale bar, 20 μm. (B) Immunoblot of FAK in the nuclear and cytoplasmic fraction of hESCs. β-tubulin serves as cytoplasmic (C) marker and NANOG as nuclear (N) enriched marker. (C) Immunoblot of FAK in the nuclear and cytoplasmic fraction of hESCs treated with DMSO or FAKi for 6 hr. Graph: densitometry. Data represent mean + SEM (n = 3 experiments). ^∗^p < 0.05. (D) Immunofluorescence images of hESCs treated with DMSO, FAKi, IgG, or MAB13 for 1 hr. Cells were co-stained with DAPI and antibodies against FAK. Arrows indicate areas with less FAK staining. Scale bar, 50 μm. (E) Immunoblot of pMDM2, p53, and β-tubulin in hESCs treated with DMSO, FAKi, IgG, or MAB13 for 6 hr. (F) Fluorescence microscopy images of hESCs treated with DMSO, FAKi, IgG, or MAB13 for 1 hr. Cells were co-stained with DAPI and antibody against p53. Scale bars, 50 μm. (G) Immunoblot of ubiquitinated p53 immunoprecipitated from hESCs treated with or without FAKi for 6 hr. See also Figure S3.

The results reported in Figures 1C and 2A link FAK inhibition with the downregulation of AKT and a subsequent apoptotic response. It has been reported that de-adhesion or kinase inhibition promotes nuclear accumulation of FAK (Lim et al., 2012), which exercises kinase-independent functions, including scaffolding MDM2 ubiquitination of p53 as an additional mechanism to support survival (Lim et al., 2008). To test whether FAK participates in this process in hESCs, we first determined its cellular location. Immunofluorescence of hESCs with total FAK antibody revealed a diffuse distribution in the cytoplasm but also in the nucleus, while FAKY397 localized at the cell surface (Figure 3A). Accordingly, FAK was repeatedly found in the nuclear fraction of hESCs (Figure 3B). After 6 hr of integrin blocking or FAK inhibition, total FAK did not accumulate in the nucleus, but was reduced in the cytoplasm and also slightly so in the nucleus (Figures 3C and S3A). Immunofluorescence also showed a widespread loss of FAK after 1 hr from integrin/FAK inhibition (Figure 3D). Moreover, inhibition of integrin/FAK deactivated pMDM2 while upregulating p53 (Figure 3E). The elevation of p53 protein was detectable already after 1 hr of treatments (Figure S3B), and we observed its increase mainly in the nucleus (Figure 3F). Furthermore, p53 upregulation appears linked to the reduced MDM2 ubiquitin ligase activity, since FAK inhibition reduces the poly-ubiquitin chains associated with p53 (Figure 3G). In conclusion, attachment of hESCs to the ECM supports FAK kinase and total protein expression, which keeps MDM2 active and p53 low, ultimately preventing a caspase-dependent anoikis.

### hESCs Avoid Anoikis by Exiting Their Undifferentiated State

**Figure 4:**
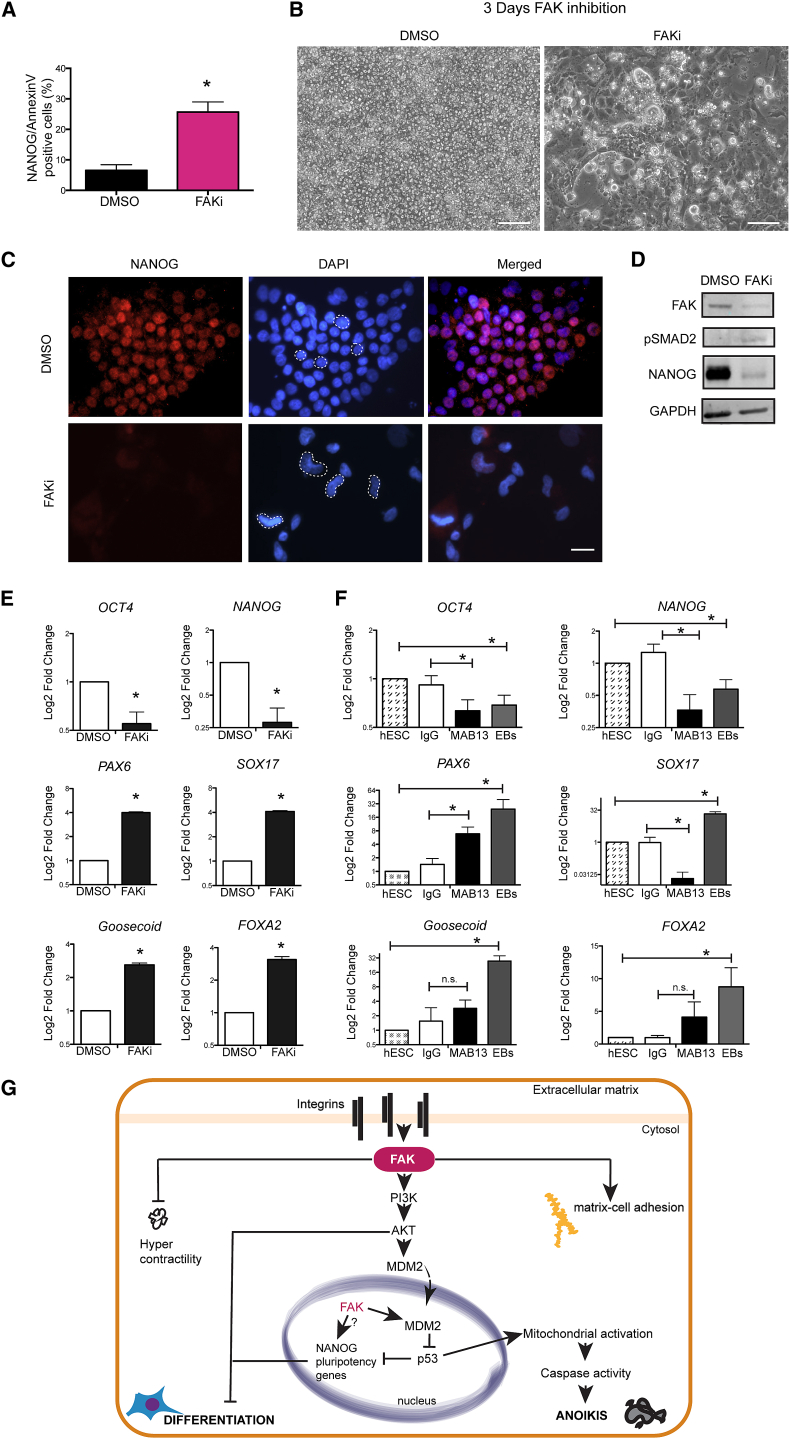
hESCs Avoid Anoikis by Exiting Their Undifferentiated State (A) Quantification of Annexin V/NANOG double-positive hESCs treated for 24 hr with DMSO or FAKi. ^∗^p < 0.05. (B) Phase images of hESCs treated with DMSO or FAKi for 3 days. Scale bar, 100 μm. (C) Immunofluorescence images of hESCs treated with DMSO or FAKi for 3 days. Cells were co-stained with DAPI and antibody against NANOG. Scale bar, 50 μm. (D) Immunoblot of FAK, pSMAD2, and NANOG in hESCs treated with DMSO or FAKi for 3 days. (E) Gene expression showing fold change for pluripotency-associated markers *NANOG* and *OCT4* and early differentiation markers *PAX6*, *SOX17*, *Goosecoid*, and *FOXA2* in hESCs treated with DMSO or FAKi for 3 days. ^∗^p < 0.05 relative to DMSO. (F) Gene expression fold change for pluripotency-associated and early differentiation markers [as in (E)] in hESCs treated with 10 μg/ml of MAB13 or IgG control for 3 days. ^∗^p < 0.05, MAB13 relative to IgG and EBs relative to hESCs; n.s., not significant. (G) Proposed model for FAK signaling in hESCs: ECM-integrin binding activates FAK, which induces PI3K upstream of AKT/MDM2 survival cascade leading to suppression of p53. In absence of FAK activity, the concomitant switch-off of AKT and elevation of p53 induces a caspase-dependent anoikis or downregulation of hESCs core genes and differentiation. Data represent mean + SEM (n = 3 experiments). See also Figure S4.

We have demonstrated that integrin signaling regulates survival of hESCs, but does it play a role in maintaining the undifferentiated stem cell state? First, we found that after 24 hr of FAKi the entire population of apoptotic hESCs retained the pluripotency-associated marker NANOG (Figure 4A). However, when hESCs were cultured in the presence of FAKi for 3 days, a subpopulation of cells that remained attached and escaped anoikis had acquired a differentiated morphology (Figures 4B and S4A). Indeed, the cells were NANOG negative and had elongated nuclei after 2 or 1 μM of FAKi (Figures 4C and S4C). Similar results were obtained on vitronectin and laminin (Figure S4D). Strikingly, FAKi-differentiated cells dramatically downregulated FAK and NANOG proteins while pSMAD2 was slightly increased (Figure 4D). At the gene-expression level, FAKi reduced both *NANOG* and *OCT4* pluripotency-associated genes (Figures 4E and S4B). In parallel, early differentiation genes were upregulated (Figure 4E). Consistent with the role of FAK downstream of integrins, blocking β1-integrin also induced differentiation in a similar fashion (Figure 4F). Integrin-blocked aggregates were comparable with embryoid bodies (EBs) in the induction of differentiation, although EBs showed a greater induction of differentiation markers at day 3 (Figure 4F), but similar to MAB13 inhibited aggregates at day 5 (Figure S4E). Together, these data show that hESCs undertake one of two routes after inhibition of integrin/FAK signaling: they die through anoikis, remaining undifferentiated, or survive but differentiate, losing characteristic hESCs morphology and markers. Our data highlight a model of hESC regulation by integrin signaling (Figure 4G) with FAK as a major transducer of integrin cues for attachment, survival, and maintenance of stem cell identity.

## Discussion

Here we shed light on the events downstream of integrin activation in hESCs and reveal in FAK the mediator of this signaling and a positive regulator of survival, adhesion, and stem cell maintenance. Indeed, hESCs respond to FAK inhibition by exiting the stem cell state through either anoikis or differentiation. We found that integrin activation in hESCs is transduced by FAKY397 to activate AKT and MDM2 and suppress p53 and caspase activation. This FAK-dependent survival pathway is consistent with that reported in adult cell types (Lim et al., 2008), but in the context of hESCs we reveal how integrin signaling supports pluripotency circuits, since AKT and p53 are well known to regulate the balance between self-renewal and differentiation (Singh et al., 2012, Jain et al., 2012). We found no evidence that the loss of FAK signaling in pluripotent cells biases differentiation to a particular lineage, but rather the resultant switch-off of PI3K/AKT and switch-on of p53 can shift hESCs out of pluripotency (Singh et al., 2012, Jain et al., 2012).

Our data confirm and extend previous work reporting FAK and AKT phosphorylation in hESCs (Miyazaki et al., 2012, Rodin et al., 2014, Wrighton et al., 2014). However, a recent paper reported that FAK is activated only upon differentiation (Villa-Diaz et al., 2016), similarly to murine ESCs where stem cell maintenance inversely correlates with integrin activation (Toya et al., 2015). This discrepancy with our and previous reports on hESCs may be explained by our data showing that the integrin signaling players are indeed active in hESCs but are only assembled into obvious focal adhesions upon differentiation. Clearly, hESCs manipulate complex integrin machinery for different purposes in different environments. For example, it was shown that ILK inhibition but not FAK inhibition increased endoderm differentiation in the presence of activin A (Wrighton et al., 2014), whereas we saw no effect of ILK on survival or differentiation when added to hESC media.

We found that hESCs require FAK for maintenance of substrate adhesion, which is consistent with its role in transmitting forces from integrins to the cytoskeleton (Huveneers and Danen, 2009). Double inhibition of FAK kinase and caspase revealed signs of cytoskeletal hypercontraction, similar to the unique and lethal hESC-response to single-cell dissociation (Ohgushi et al., 2010), but also visible in groups of mutually adhering cells. Thus, our results suggest that integrin signaling is an essential yet distinct cue from cell-cell adhesion for suppression of cytoskeleton contraction and apoptosis. However, integrin signaling may crosstalk with cell-cell adhesion. Indeed, we observed formation of cell aggregates after blocking β1-integrin or FAK knockdown.

Finally, we discovered that hESCs possess a nuclear pool of FAK that does not accumulate but reduces after FAK inhibition, unlike in adult cells (Lim et al., 2012). We propose that hESCs utilize non-canonical FAK mechanisms to quickly respond to defective adhesion, which could involve undiscovered roles for nuclear FAK in the context of pluripotent cells.

In conclusion, this study shows that the ECM exerts extensive control over feeder-free hESCs via a FAK-dependent cascade linking integrins to intrinsic regulatory players, supporting survival and maintenance of stem cell identity. This study advances our understanding of the underlying integrin signaling controlling hESCs, of particular importance for their culture on ECM for both basic and translational research.

## Experimental Procedures

### Cell Culture

hESCs HUES1 (Harvard University), MAN5 (North West Embryonic Stem Cell Center), Shef-1 (University of Sheffield), and hiPSCs Sendai J (Neusentis) were cultured in defined mTSER1 (Life Technologies) medium on 50 μg/mL fibronectin as previously described (Baxter et al., 2009). Cells were passaged with TryPLE express (Gibco) at a ratio of 1:3. HUES1 was used as lead line throughout the study, MAN5 was used when specified, and Shef1 and SendaiJ to confirm compound screening. For selected experiments, hESCs were plated on 5 μg/mL of vitronectin-N (Life Technologies) or laminin 521 (Biolamina).

### Integrin Blocking

hESCs were dissociated and seeded at a density of 0.7 × 10^4^ cells/cm^2^ in hESC media supplemented with 10 μg/mL MAB13 anti β1-integrin antibody (M. Humphries, University of Manchester) or rat immunoglobulin G (IgG) isotype (Sigma) and plated on fibronectin. For cells cultured on vitronectin, αVβ5-integrin was inhibited with 10 μg/ml MAB1916z blocking antibody (Millipore) or mouse IgG isotype (Sigma). For antibody-free and EB controls, hESCs were seeded at the same density on either fibronectin-coated or non-adherent tissue-culture plates, respectively.

### Compound Screening

Cells were prepared 24 hr before the assay by passaging in presence of Y-27632 (10 μM) (Sigma-Aldrich) to a fibronectin-coated 96-well plate (Cell-BIND, Corning) at a density of 2 × 10^4^ cells/well. Cells were treated in triplicate with FAK or AKT inhibitors, at the concentrations indicated in the figures, added to mTESR1 medium, and incubated for 5 hr at 37°C. The ApoToxGlo Triplex assay (Promega) was used according to the manufacturer's instructions. Signals were analyzed with a SpectraMax M5 reader (Molecular Devices).

### siRNA Knockdown

hESCs were treated with 10 μM Y27632 (Sigma-Aldrich) for 1 hr and nucleofected with Amaxa Nucleofector following the manufacturer's instructions (Lonza), with target-specific FAK siRNA (sc-29310, Santa Cruz Biotechnology) at a final concentration of 200 nM, β-2-microglobulin siRNA control (Life Technologies), or pmaxGFP control vector (Lonza). Medium was changed the following day and experimental analysis performed 66 hr later.

### Statistics

Data were analyzed with Student's *t*-test using GraphPad Prism. p values of <0.05 were considered significant.

## Author Contributions

L.V. designed and performed the experiments; M.B. performed integrin analysis; B.I. performed the Src and integrin αVβ5 experiments; P.W. supervised the compound screening; S.J.K. designed and supervised the study; L.V. and S.J.K. co-wrote the manuscript.

## Supplemental Information

Document S1. Supplemental Experimental Procedures, Figures S1–S4, and Table S1

Document S2. Article plus Supplemental Information
