# Supplementary material for: Integrin-Associated Focal Adhesion Kinase Protects Human Embryonic Stem Cells from Apoptosis, Detachment, and Differentiation
Source: Stem Cell Reports. 2016 Aug 9;7(2):167–76. doi: 10.1016/j.stemcr.2016.07.006 (PMC4983079; doi:10.1016/j.stemcr.2016.07.006)
Supplement: Document S1. Supplemental Experimental Procedures, Figures S1–S4, and Table S1 [file mmc1.pdf]

Stem Cell Reports, Volume *7*

Supplemental Information

Integrin-Associated Focal Adhesion Kinase Protects Human Embryonic Stem Cells from Apoptosis, Detachment, and Differentiation

Loriana Vitillo, Melissa Baxter, Banu Iskender, Paul Whiting, and Susan J. Kimber

SUPPLEMENTAL FIGURES

FIGURE S1

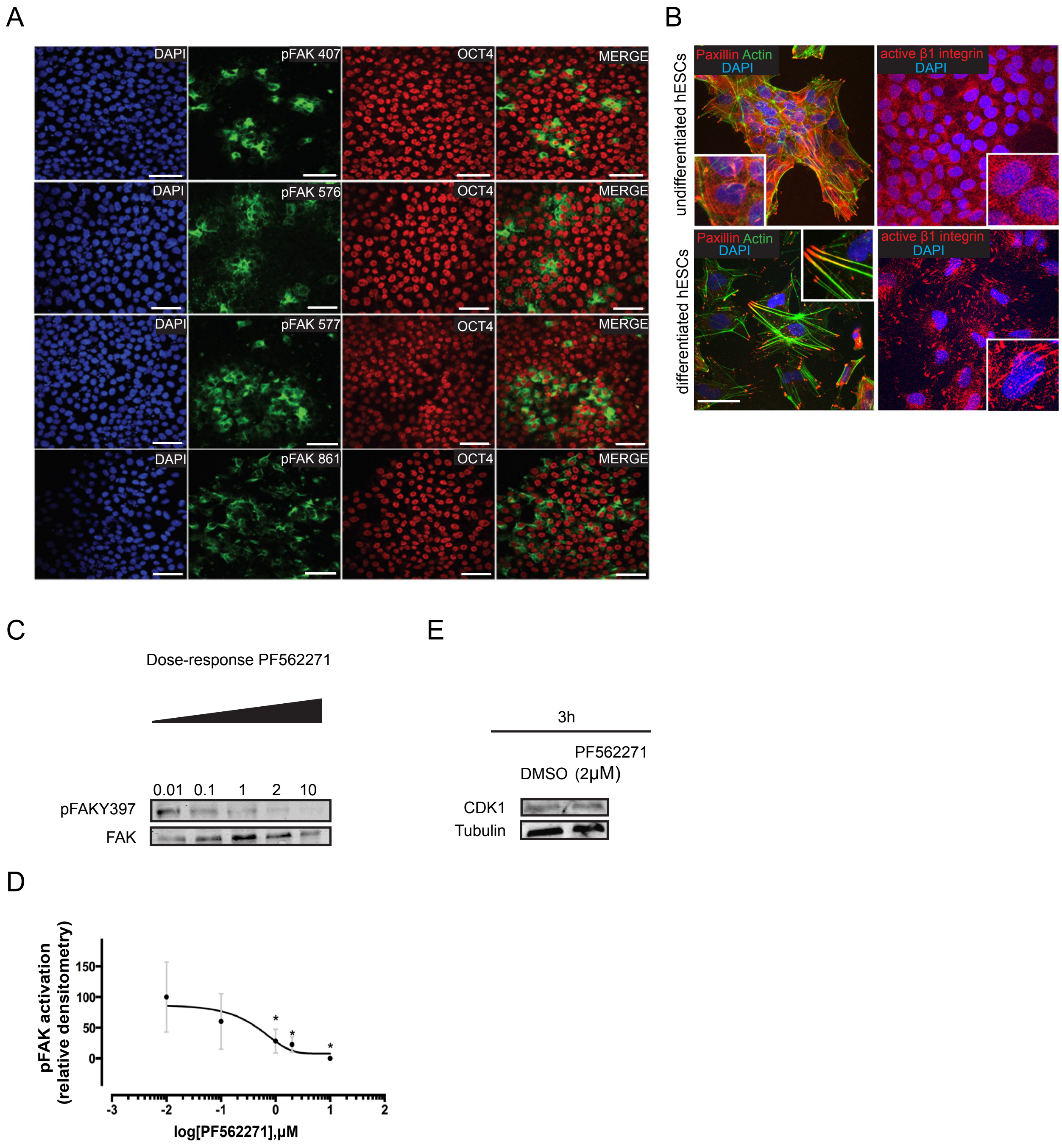

Figure S1. Matrix-Integrin Binding Activates FAK Signaling Upstream of AKT (Related to Figure 1)

(A) Immunofluorescence images of hESCs cultured on fibronectin and stained with antibodies against OCT4 and pFAKY407, pFAKY576, pFAKY577 or pFAKY861. Scale bars 50 µm
(B) Immunofluorescence image of undifferentiated and differentiated hESCs cultured on fibronectin and stained with antibodies against Paxillin, Actin and active β1-integrin. For differentiation, cells were grown in hESCs media (Baxter et al., 2009) without NT4 and AA for 7 days. Scale bar 50 µm
(C) Immunoblot of pFAKY397 and FAK after increasing concentrations of PF562271 inhibitor added for 1 h.
(D) Dose-response curve of pFAKY397 relative to PF562271 concentration added for 1 h. Average of 3 independent experiments. *indicates $p<0.05$.
(E) Immunoblot of CDK1 and β-tubulin after 3 hr of 2 µM PF562271.

Data represented as mean +SEM

FIGURE S2

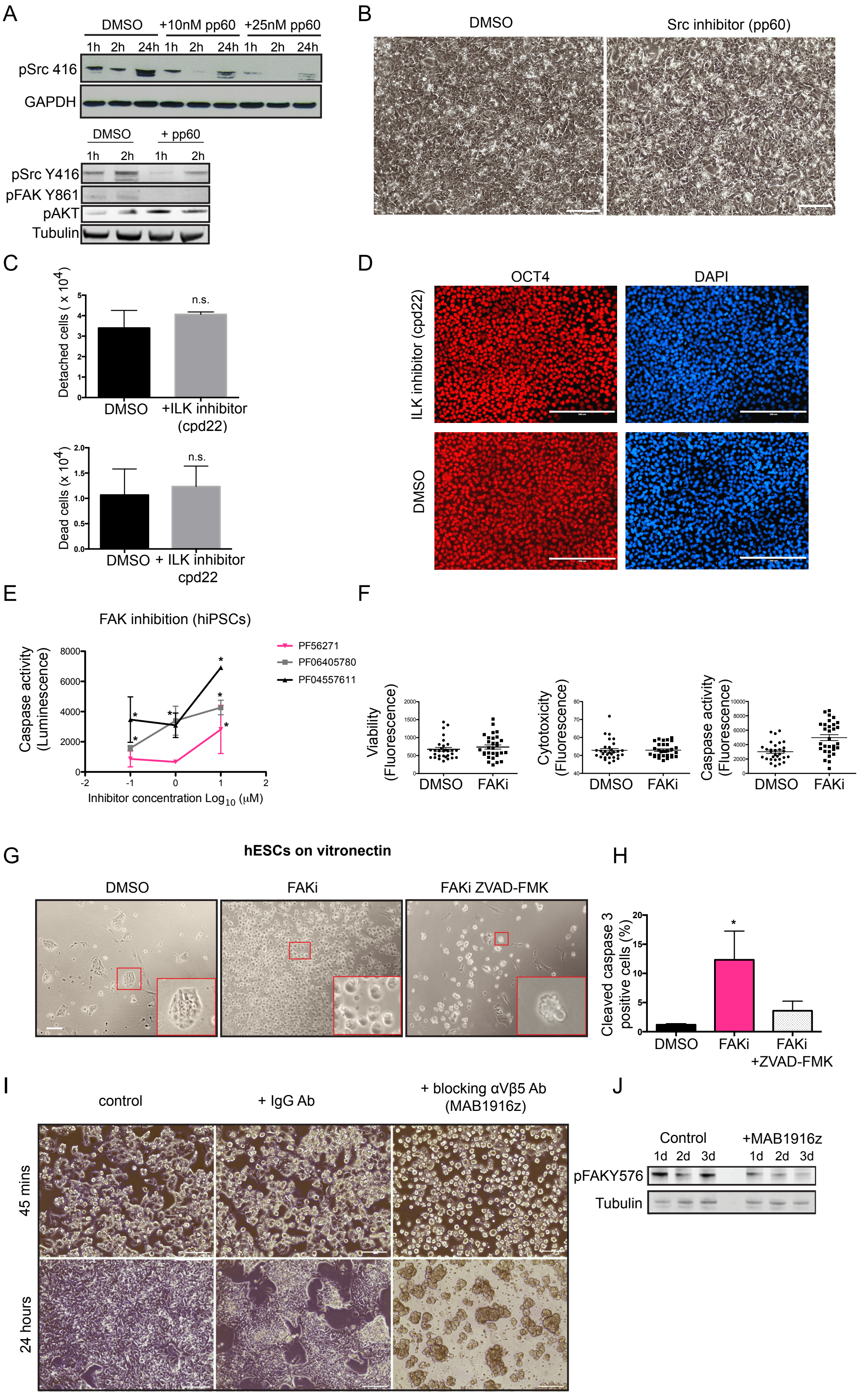

A
DMSO
+10nM pp60
+25nM pp60
1h 2h 24h 1h 2h 24h 1h 2h 24h
pSrc 416
GAPDH
DMSO
+ pp60
1h 2h 1h 2h
pSrc Y416
pFAK Y861
pAKT
Tubulin
B
DMSO
Src inhibitor (pp60)
C
Detached cells (x 10^4)
n.s.
DMSO
+ILK inhibitor (cpd22)
Dead cells (x 10^4)
n.s.
DMSO
+ ILK inhibitor cpd22
D
OCT4
DAPI
ILK inhibitor (cpd22)
DMSO
E
FAK inhibition (hiPSCs)
Caspase activity (Luminescence)
PF56271
PF06405780
PF04557611
Inhibitor concentration Log$_{10}$ (µM)
F
Viability (Fluorescence)
Cytotoxicity (Fluorescence)
Caspase activity (Fluorescence)
DMSO
FAKi
G
hESCs on vitronectin
DMSO
FAKi
FAKi ZVAD-FMK
H
Cleaved caspase 3 positive cells (%)
DMSO
FAKi
FAKi +ZVAD-FMK
I
control
+ IgG Ab
+ blocking αVβ5 Ab (MAB1916z)
45 mins
24 hours
J
Control
+MAB1916z
1d 2d 3d 1d 2d 3d
pFAKY576
Tubulin

Figure S2. Inhibition of FAK Signaling Induces Cell Blebbing and a Caspase-Dependent Anoikis (Related to Figure 2)

(A) Top: Immunoblot of pSrc416 and GAPDH in hESCs treated with DMSO or Src inhibitor pp60 at 10nM or 25nM for 1h, 2h or 24hr. Bottom: Immunoblot of pSrc416, pFAKY861, pAKT and β-tubulin in hESCs treated with DMSO or pp60 (25nM) for 1 and 2 hr.
(B) Phase images of hESCs plated on fibronectin for 24 hr in presence of Src inhibitor pp60 (25 nM) or DMSO control. Scale bars 100 μm
(C) Quantification of cell detachment (cell in suspension) and survival (dead cells stained positive with trypan blue) in hESCs treated for 24 hr with 0.4 μM of ILK inhibitor cpd22. (n=3 independent experiments).
(D) Immunofluorescence images of hESCs treated with DMSO or 0.4 μM cpd22 for 3 days and stained with antibody against OCT4.
(E) Caspase activity in hiPSCs treated for 5 hr with the indicated concentrations of FAK inhibitors. *indicates $p<0,05$ (n=3 independent experiments).
(F) Scatter plots of viability, cytotoxicity and caspase activity measured in parallel in hiPSCs treated with DMSO or 2 μM of PF562271 for 5 hr. Each dot represents a well of a 96-well plate.
(G) Phase images of hESCs cultured on vitronectin substrate and treated for 24 hr with DMSO, FAKi only or with Z-VAD-FMK. Inserts from left to right show: hESCs colony, cells undergoing anoikis and cell blebbing. Scale bar 50 μm
(H) Cleaved-caspase 3 expression in hESCs cultured on vitronectin and treated with DMSO, FAKi only or with Z-VAD-FMK for 24 hr. *indicates $p<0.05$ relative to DMSO (n=3 independent experiments).
(I) Phase images of hESCs replated on vitronectin for 45 mins or 24 hr in presence of 10 μg/ml of MAB1916z or mouse IgG control versus untreated control. Scale bars 100 μm.
(J) Immunoblot of pFAKY576 or β-tubulin in hESCs cultured on vitronectin from 1 to 3 days (3d) with 10 μg/ml of IgG control or MAB1916z.

Data represented as mean +SEM

FIGURE S3

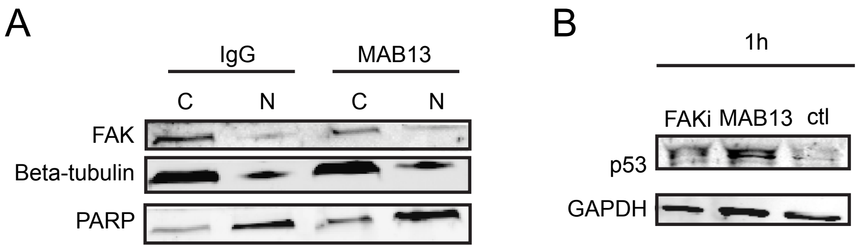

Figure S3. FAK Localizes in the Nucleus of hESCs and Regulates MDM2/p53 Levels during Anoikis (Related to Figure 3)

(A) Immunoblot of FAK in the nuclear and cytoplasmic fraction of hESCs treated with IgG control or MAB13 for 6 hr. β-tubulin serves as cytoplasmic marker (C) and PARP as nuclear marker (N).
(B) Immunoblot of p53 and GAPDH in control hESCs versus cells treated with FAKi or MAB13 for 1 hr.

FIGURE S4

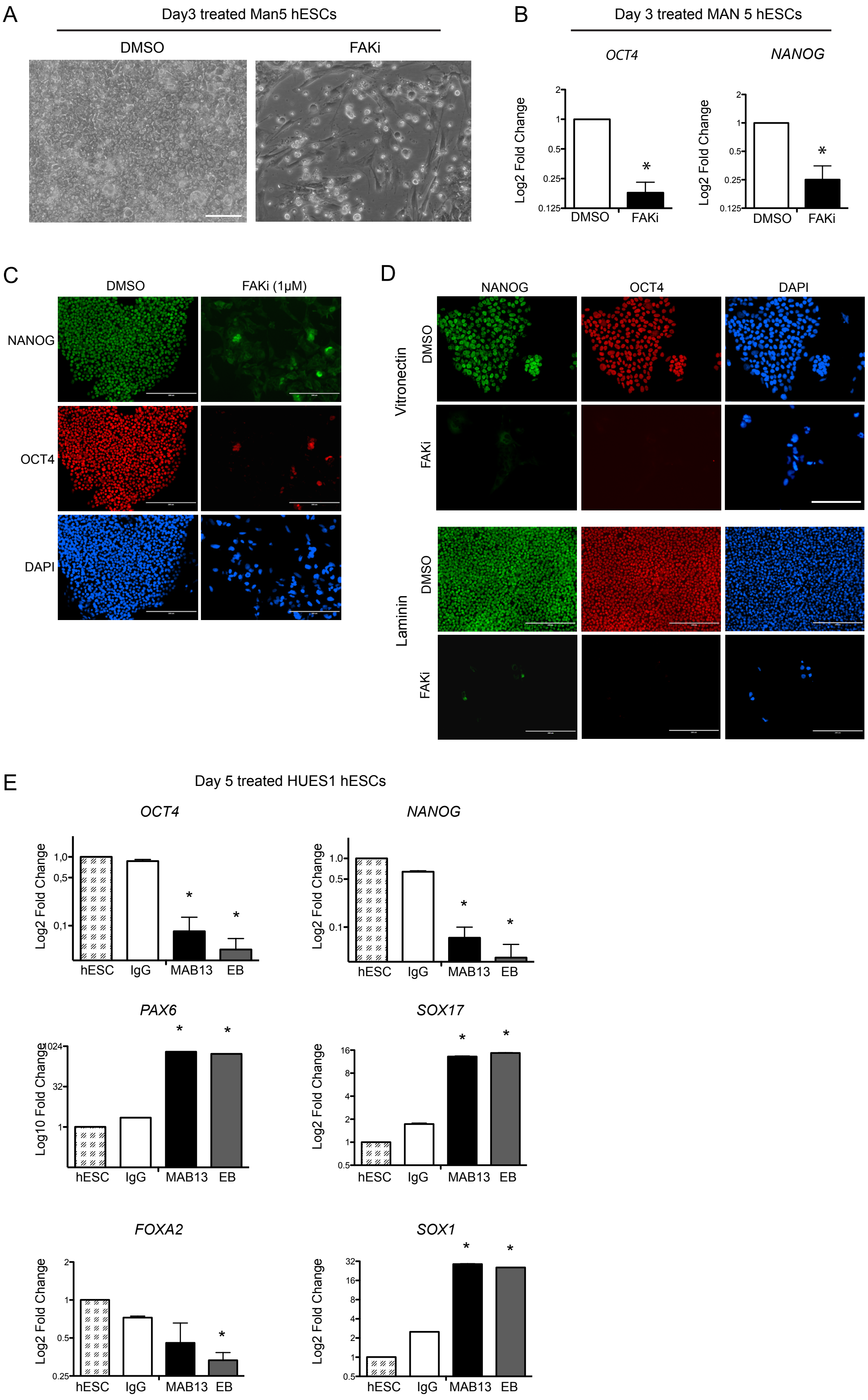
A
Day3 treated Man5 hESCs
DMSO
FAKi
B
Day 3 treated MAN 5 hESCs
OCT4
NANOG
Log2 Fold Change
DMSO
FAKi
C
DMSO
FAKi (1μM)
NANOG
OCT4
DAPI
D
NANOG
OCT4
DAPI
Vitronectin
Laminin
DMSO
FAKi
E
Day 5 treated HUES1 hESCs
OCT4
NANOG
PAX6
SOX17
FOXA2
SOX1
Log2 Fold Change
Log10 Fold Change
hESC
IgG
MAB13
EB

Figure S4. hESCs Avoid Anoikis by Exiting their Undifferentiated State (Related to Figure 4)

(A) Phase images of hESCs MAN5 treated with DMSO or 2 µM of PF562271 (FAKi) for 3 days. Scale bar 100 µm.
(B) Gene expression fold-change of pluripotency markers *NANOG* and *OCT4* in hESC MAN5 treated with DMSO or FAKi for 3 days. * indicates p<0,05 (n=3 independent experiments).
(C) Immunofluorescence images of hESCs cultured on fibronectin and treated with DMSO or 1 µM PF562271 for 3 days. Cells were costained with DAPI and antibodies against NANOG and OCT4. Scale bars 200 µm.
(D) Immunofluorescence images of hESCs cultured on vitronectin or laminin 521 and treated with DMSO or 2 µM PF562271 for 3 days. Cells were costained with DAPI and antibodies against NANOG and OCT4. Scale bars 200 µm.
(E) Gene expression fold-change of pluripotency markers *NANOG*, *OCT4* and differentiation markers *PAX6*, *SOX17*, *FOXA2* and *SOX1* in hESC HUES1 treated with DMSO, FAKi, 10µg of MAB13 or rat IgG for 5 days. * indicates p<0,05. MAB13 relative to IgG, EB relative to hESCs (n=3 independent experiments)

Data represented as mean +SEM

SUPPLEMENTAL EXPERIMENTAL PROCEDURES

Apoptosis assay and Flow Cytometry:
Apoptosis measurements were performed using the Annexin V-PE and 7-AAD kit (BD Pharmigen) and acquired on a Lab Quanta SC flow cytometer (Beckman Coulter). For Annexin V/NANOG double staining, samples were pre-stained with Annexin V for 15 mins and washed 3 times with PBS 1X prior to fixation. For intracellular staining, cells were fixed in 4% paraformaldehyde at room temperature and permeabilized in 70% MeOH at 4°C. Samples were probed with Nanog-PE or AlexaFluor 488 Isotype control (both from BD Pharmigen) for 30 mins and analysed. Data were analysed using Summit 4.3 (Beckman Coulter).

Immunofluorescence:
hESCs were fixed in 4% paraformaldehyde, blocked with 10% serum matching the species of the secondary antibody and permeabilised with 0.1% TritonX-100. Cells were incubated with primary antibodies dilutions containing 1% serum overnight at 4°C. Cells were washed and incubated with secondary antibodies conjugated with Alexa fluorophores (Invitrogen) and nuclei stained with DAPI (Sigma). Images were taken with Olympus 1X71 inverted microscope (Olympus) with a Q-imaging Retiga SRV camera and processed using QCapture Pro (QImaging) and ImageJ 64.

Widefield restoration microscopy with Delta Vision:
hESCs were grown and fixed on glass-bottom culture dishes (35.0/10mm Grainer Bio-one) coated with fibronectin. Images were acquired on a Delta Vision Olympus 1X71 microscope (Applied Precision) restoration microscope using a 100x/140 Uplan S Apo objective and the Sedat Quad filter set. The images were collected using a Coolsnap HP (Photometrics) camera with a Z optical spacing of 0.2 μm.

Antibodies and reagents:
Immunofluorescence antibodies were used against: Paxillin (BD Bioscience), F-Actin (Fluorescein phalloidin, Invitrogen), activated β1-integrin 12g10 (gift from prof. M.Humphries, Wellcome Matrix Centre, The University of Manchester), NANOG (R&D Systems); OCT4 (BD Biosciences); FAK clone 4.47 (Millipore); pFAKY397, pFAKY407, pFAKY576, pFAKY577 and pFAKY861 (Invitrogen); p53 (ab131442, Abcam), Cleaved-caspase3 (Asp175, Cell Signalling).
Western blotting antibodies were against: FAK, pFAKY397, pFAK576, pSrc416, pAKTSer473, p53, pMDM2, NANOG (all from Cell Signalling); pFAK861 (Invitrogen); CDK1 (R&D); GAPDH 71.1 (Sigma Aldrich); AKT (Invitrogen); pSMAD2 (Millipore); Multiubiquitin Chain MoAb (clone FK2, Cayman chemical); β-tubulin (Li-COR). Secondary antibodies: IRDye 680LT Goat anti-Mouse IgG; IRDye 800CW Goat anti-Rabbit IgG (Li-COR), IRDye 680LT Donkey anti-Goat IgG.
Small molecules: FAK inhibitors PF-562271 (Roberts et al., 2008), PF-06405780 (Bunnage et al., 2011), PF-04557611 (Lovering et al., 2012); AKT inhibitors PF-05094152 (Heerding et al., 2008), PF-04907559 (Heerding et al., 2007) and PF-04943582 (Zhu et al., 2007) were all provided by Pfizer. ILK inhibitor cpd22 from Calbiochem. Src inhibitor pp60 (521-533) from Tocris. Caspase inhibitor Z-VAD-FMK from Abcam.

Protein extraction and immunoblotting:
Adherent cells were lysed on ice in RIPA buffer (Sigma) with added protease and phosphatase inhibitors (ROCHE). Protein extracts were quantified using the BCA protein detection kit (Thermo Scientific). Equal protein amounts were separated on 12% poly-acrylamide gel and transferred to a nitrocellulose membrane (Wathman). Membranes were blocked in Odyssey buffer (Li-COR), incubated overnight at 4°C with primary antibody, washed and incubated with IRDye conjugated secondary antibodies for 2 hr at room temperature. Membranes were scanned with the Odyssey detection system (Li-COR).

Subcellular fractionation:
hESCs pellets were lysed in NER-PER extraction reagents (Thermo Scientific). Cell pellet were resuspended in 200 μl of CERI buffer plus protease and phosphatase inhibitors (ROCHE) for 10 mins on ice. CERII buffer was added to the solution, vortexed for 15 mins, incubated 2 mins on ice, vortexed 5 sec and spun at 500 g for 7 mins. Cytoplasmic proteins were collected from the supernatant. The remaining nuclear pellet was washed twice in PBS, spun at 500 g for 7 min and incubated in 50 μl of NER buffer for 40 mins on ice, vortexed every 10 mins. Nuclear proteins were collected in the supernatant after a 16,000 g centrifugation for 10 mins at 4°C.

Immunoprecipitation:
hESCs pellet corresponding to 5 x 10^6 cells was used for each immunoprecipitation condition following the p53 immunocapture kit (ab154470, Abcam). For each immunoprecipitation, 10 μl of p53 beads slurry were used in a 1.5 ml tube incubated under rotation for 16 hr at 4°C.

Real-time PCR:
Total RNA was isolated using the RNeasy Mini Kit (Qiagen) and converted to cDNA with the M-MLV reverse transcriptase (Promega). For qPCR mastermixes, power SYBR Green Master Mix (Applied Biosystems) was used and plates were run using the CFX96 Real-Time System (Bio-Rad). mRNA levels were normalized to GAPDH expression and relative expression calculated as fold-change from the untreated control.

Table S1. Primer set used in this study, Related to Figure 4

GENE	FORWARD	REVERSE
OCT4	AGACCATCTGCCGCTTTGAG	GCAAGGGCCGCAGCTT
NANOG	GGCTCTGTTTTGCTATATCCCCTAA	CATTACGATGCAGCAAATACAAGA
GAPDH	ATGGGGAAGGTGAAGGTCG	TAAAAGCAGCCCTGGTGACC
PAX6	CTGGCTAGCGAAAAGCAACAG	CCCGTTCAACATCCTTAGTTTATCA
SOX17	AGAGATTTGTTTCCCATAGTTGGATT	TGTTTTGGGACACATTCAAAGCT
SOX1	GCGGTAACAACTACAAAAAACTTGTAA	GCGGAGCTCGTCGCATT
FOXA2	TTCAGGCCCGGCTAACTCT	AGTCTCGACCCCCACTTGCT
Goosecoid	GATGCTGCCCTACATGAACGT	GACAGTGCAGCTGGTTGAGAAG

SUPPLEMENTAL REFERENCES

Baxter, M.A., Camarasa, M.V., Bates, N., Small, F., Murray, P., Edgar, D., and Kimber, S.J. (2009). Analysis of the distinct functions of growth factors and tissue culture substrates necessary for the long-term self-renewal of human embryonic stem cell lines. Stem Cell Res 3, 28-38.

Bunnage, M.E., Cook, A.S., Cui, J.J., Dack, K.N., Deal, J.G., Gu, D., He, M., Johnson, P.S., Johnson, T.W., Le, P.T.Q., et al. (2011). Preparation of heterocyclic derivatives such as 2-aminopyridine and 3-aminopyridazine derivatives as anaplastic lymphoma kinase (ALK) inhibitors for the treatment of diseases. PCT Int. Appl. WO 2011138751 A2 20111110.

Heerding, D.A., Clark, T.J., Leber, J.D., Safonov, I.G. (2007). 1H-inidazo[4,5-c]pyridin-2-yl derivatives as inhibitors of AKT activity and their therapeutic uses. PCT Int. Appl. WO 2007058850 A2 20070524.

Heerding, D.A., Rhodes, N., Leber, J.D., Clark, T.J., Keenan, R.M., Lafrance, L.V., Li, M., Safonov, I.G.,Takata, D.T., Venslavsky, J.W., et al. (2008). Identification of 4-(2-(4-aminso-1,2,5-oxadiazol-3-yl)-1-ethyl-7-{[(3S)-3-piperidinylmethyl]oxy}-1H- imidazo[4,5-c]pyridin-4-yl)-2-methyl-3-butyn-2-ol (GSK690693), a novel inhibitor of AKT kinase. J Med Chem 51, 5663-5679.

Lovering, F., McDonald, J., Whitlock, G.A., Glossop, P.A., Phillips, C., Bent, A., Sabnis, Y., Ryan, M., Fitz, L.,Lee, J., et al. (2012). Identification of type-II inhibitors using kinase structures. Chem Biol Drug Des 80, 657-664.

Roberts, W.G., Ung, E., Whalen, P., Cooper, B., Hulford, C., Autry, C., Richter, D., Emerson, E., Lin, J., Kath, J., et al. (2008). Antitumor activity and pharmacology of a selective focal adhesion kinase inhibitor, PF-562,271. Cancer Res 68, 1935-1944.

Zhu, G.D., Gandhi, V.B., Gong, J., Thomas, S., Woods, K.W., Song, X., Li, T., Diebold, R.B., Luo, Y., Liu, X., etal. (2007). Syntheses of potent, selective, and orally bioavailable indazole-pyridine series of protein kinase B/Akt inhibitors with reduced hypotension. J Med Chem 50, 2990-3003.
