# Supplementary material for: Integrin-Associated Focal Adhesion Kinase Protects Human Embryonic Stem Cells from Apoptosis, Detachment, and Differentiation
Source: Stem Cell Reports. 2016 Aug 9;7(2):167–76. doi: 10.1016/j.stemcr.2016.07.006 (PMC4983079; doi:10.1016/j.stemcr.2016.07.006)
Supplement: Document S2. Article plus Supplemental Information [file mmc2.pdf]

Stem Cell Reports
Report

OPEN ACCESS

Integrin-Associated Focal Adhesion Kinase Protects Human Embryonic Stem Cells from Apoptosis, Detachment, and Differentiation

Loriana Vitillo,[1,3] Melissa Baxter,[1,4] Banu Iskender,[1,5] Paul Whiting,[2,6] and Susan J. Kimber[1,*]
[1]North West Embryonic Stem Cell Centre, Faculty of Life Sciences, University of Manchester, Manchester M13 9PT, UK
[2]Pfizer Neusentis, The Portway Building, Granta Park, Cambridge CB21 6GS, UK
[3]Present address: Wellcome Trust-Medical Research Council Stem Cell Institute, Anne McLaren Laboratory for Regenerative Medicine, University of Cambridge, Cambridge CB2 0SZ, UK
[4]Present address: School of Medicine and Dentistry, University of Central Lancashire, Preston PR1 2HE, UK
[5]Present address: Betul-Ziya Eren Genome and Stem Cell Center, Erciyes University, Kayseri 38039, Turkey
[6]Present address: Alzheimer's Research UK UCL Drug Discovery Institute, University College London, London WC1E 6BT, UK
*Correspondence: sue.kimber@manchester.ac.uk
http://dx.doi.org/10.1016/j.stemcr.2016.07.006

SUMMARY

Human embryonic stem cells (hESCs) can be maintained in a fully defined niche on extracellular matrix substrates, to which they attach through integrin receptors. However, the underlying integrin signaling mechanisms, and their contribution to hESC behavior, are largely unknown. Here, we show that focal adhesion kinase (FAK) transduces integrin activation and supports hESC survival, substrate adhesion, and maintenance of the undifferentiated state. After inhibiting FAK kinase activity we show that hESCs undergo cell detachment-dependent apoptosis or differentiation. We also report deactivation of FAK downstream targets, AKT and MDM2, and upregulation of p53, all key players in hESC regulatory networks. Loss of integrin activity or FAK also induces cell aggregation, revealing a role in the cell-cell interactions of hESCs. This study provides insight into the integrin signaling cascade activated in hESCs and reveals in FAK a key player in the maintenance of hESC survival and undifferentiated state.

INTRODUCTION

Human embryonic stem cells (hESCs) are pluripotent stem cells that exhibit epithelial-like features resembling the epiblast epithelium of the post-implantation embryo (Nichols and Smith, 2009). Similarly to epithelial cells, hESCs are dependent on E-cadherin-mediated cell-cell contacts and anchorage to the extracellular matrix (ECM) via integrin receptors (Ohgushi et al., 2010; Braam et al., 2008). Various studies have established the efficacy of integrin engagement with ECM substrates in supporting hESC self-renewal and pluripotency (Braam et al., 2008; Baxter et al., 2009; Miyazaki et al., 2012; Soteriou et al., 2013; Rodin et al., 2014). However, the specific nature and role of downstream signaling from integrins in hESCs remains largely unexplored.

One of the key functions of the ECM in epithelial cells is to prevent a common form of apoptosis, anoikis, or "homelessness" of cells that have lost contact with the matrix (Frisch and Francis, 1994). Anoikis is executed via the mitochondrion and results in activation of caspase downstream of integrin-associated pathways (Gilmore et al., 2000). ECM-integrin interaction initiates signaling, promoting the assembly of cytoplasmic scaffold and kinase proteins at focal adhesions near active integrin clusters (Giancotti and Ruoslahti, 1999). Focal adhesion kinase (FAK), a protein tyrosine kinase, is one of the principal integrin signaling regulators, containing three domains: the protein 4.1, ezrin, radixin, moesin (FERM) domain, the kinase domain, and the focal adhesion targeting domain (Frame et al., 2010). Upon integrin activation FAK localizes at the adhesion site where structural changes displace the inhibitory FERM, allowing autophosphorylation of the Tyr397 (Y397) site, leading to the activation of its intrinsic kinase function and the formation of docking sites for multiple downstream signaling molecules (Frame et al., 2010). Several signaling players directly interact with the Y397 site, e.g., Src, which in turn phosphorylates FAK, promoting further activation, or p130Cas, Grb2, and phosphatidylinositol 3-kinase (PI3K), involved in controlling cytoskeletal rearrangements, cell cycle, and survival (Parsons, 2003). FAK is crucial in preventing anoikis through direct activation of PI3K, via the Y397 site, in turn promoting the pro-survival AKT cascade (Gilmore et al., 2000; Xia et al., 2004). FAK can also leave focal adhesions and act in a kinase-independent manner by localizing in the nucleus where the FERM scaffolds the AKT target MDM2 for ubiquitination of pro-apoptotic p53, leading to its protein degradation (Lim et al., 2008).

Among the repertoire of integrins, the β1-integrin subunit mediates the attachment of hESCs to fibronectin via the α5β1 heterodimer (Baxter et al., 2009), as well as other commonly used ECM (Braam et al., 2008). Although hESCs cultured on ECM have been shown to express active FAK and AKT (Miyazaki et al., 2012; Rodin et al., 2014; Wrighton et al., 2014), the functional contribution of the FAK pathway to hESCs has not been dissected.

This is an open access article under the CC BY license (http://creativecommons.org/licenses/by/4.0/).

Here, we show that integrin activation in hESCs is transduced by FAK to regulate adhesion and prevent the onset of anoikis or differentiation via an AKT/MDM2/p53 cascade. Together, our results reveal a critical role for FAK in the control of hESC fate, as a mediator of integrin signaling crosstalk with key hESC regulatory players.

RESULTS

Matrix-Integrin Binding Activates FAK Signaling Upstream of AKT

To characterize integrin signaling in hESCs cultured on fibronectin, we investigated FAK activation. Immunofluorescence analysis of phosphorylation sites marking FAK activity showed widespread expression of the autophosphorylation Y397 site, induced upon integrin engagement in OCT4-positive cells (Figure 1A). Other phosphorylated residues, created by Src kinase binding to FAKY397 during adhesome assembly, were expressed in a small proportion of cells (Figure S1A) showing that hESCs display active FAK signaling. Importantly, hESCs express high levels of active β1-integrin and the focal adhesion marker paxillin but in a diffuse or punctate distribution, while upon differentiation focal adhesions are visible (Figure S1B). Next, we asked whether FAKY397 is a transducer of fibronectin/β1-integrin binding. hESCs grown on fibronectin had active FAKY397 and its downstream PI3K target AKT Ser473 (S473) (Figure 1B). Conversely, plating hESCs on a non-integrin-activating substrate, Poly-L-Lysine, or blocking β1-integrin selectively with antibody (MAB13), which we previously showed induces hESC detachment from fibronectin (Baxter et al., 2009), reduced FAK and AKT activity (Figures 1C and 1D). We then applied a selective FAK inhibitor, PF562271, which reduced pFAKY397 in a dose-dependent manner (Figures S1C and S1D). Pharmacological inhibition of 80% of FAKY397 with PF562271 at 2 μM (FAKi) was comparable with blocking β1-integrin in reduction of FAK activity (Figures 1C and 1D) without affecting the potential off-target CDK1 (Figure S1E). Moreover, after FAK inhibition there was a reduction of pAKT similar to that seen following integrin inhibition or on Poly-L-Lysine (Figures 1C and 1D). In summary, our data indicate that integrin engagement in hESCs is transduced through FAKY397 and its downstream kinase, AKT (Figure 1E).

Inhibition of FAK Signaling Induces Cell Blebbing and a Caspase-Dependent Anoikis

Autophosphorylation of FAK is crucial for the transduction of survival signals by recruiting PI3K that in turn induces the AKT cascade (Xia et al., 2004). Since our data suggested that FAKY397 transduces integrin activation to AKT, we next determined whether FAK kinase activity supports survival of hESCs. hESCs responded to FAK kinase block with PF562271 by detaching from the matrix (Figure 2E) and undergoing apoptosis in a dose-dependent manner (Figure 2A). At the same time, we excluded the possibility that the FAK/Src complex mediated survival, since inhibition of Src did not induce cell detachment and dephosphorylation of AKT even if its target FAKY861 was decreased (Figures S2A and S2B). Similarly, inhibition of the integrin-associated pseudokinase ILK, reported to target AKT in differentiating hESCs (Wrighton et al., 2014), did not affect cell attachment or survival (Figure S2C). Furthermore, inhibition of ILK did not affect pluripotency markers over time (Figure S2D).

To further confirm that the inhibition of FAK kinase is responsible for apoptosis of hESCs, we tested in parallel two other selective FAK inhibitors in both hESCs and human induced pluripotent stem cells (hiPSCs) with a high-throughput assay that measures early caspase activation. All tested FAK inhibitors induced caspase activity in proportion to dose in both hESCs (Figure 2B) and hiPSCs (Figure S2E). In addition, selective inhibitors of AKT induced caspase activity in a similar manner (Figure 2C), supporting AKT as an effector of FAK. Finally, by measuring together caspase activity, viability, and cytotoxicity after FAKi, we found that caspase activity was induced without non-specific cytotoxicity (Figure S2F).

To validate whether FAK-dependent apoptosis relies on caspase activity, we applied the caspase inhibitor Z-VAD-FMK to hESCs treated with FAKi. Immunostaining confirmed that FAKi induced cleaved caspase-3 expression that was inhibited by Z-VAD-FMK (Figure 2D). Floating dead cells, normally present after FAKi, were abolished by Z-VAD-FMK but instead, attached single and groups of hESCs, with prominently blebbing membranes, were observed (Figure 2E). The effects of FAKi on survival, cell blebbing, and caspase activation were confirmed on vitronectin (Figures S2G and S2H).

Cell blebbing is an indicator of cytoskeletal contraction, commonly a result of caspase-3 cleavage of ROCK during apoptosis, leading to increased contractility (Coleman et al., 2001). Our data indicate that FAK inhibition induced cell blebbing independently of caspase, a unique mechanism reported in hESCs after cell-cell dissociation (Ohgushi et al., 2010), but also observed by us in groups of cells. However, caspase activity is required to complete detachment from the ECM, a key feature of anoikis. Moreover, Z-VAD-FMK also rescued FAKi-dependent early (Annexin V) and late (Annexin V/7-AAD) apoptosis, increasing the proportion of live cells, suggesting a suppression of hESC caspase-dependent turnover (Figure 2F). Overall, these results show that FAK kinase activity is required to suppress a caspase-dependent anoikis in hESCs.

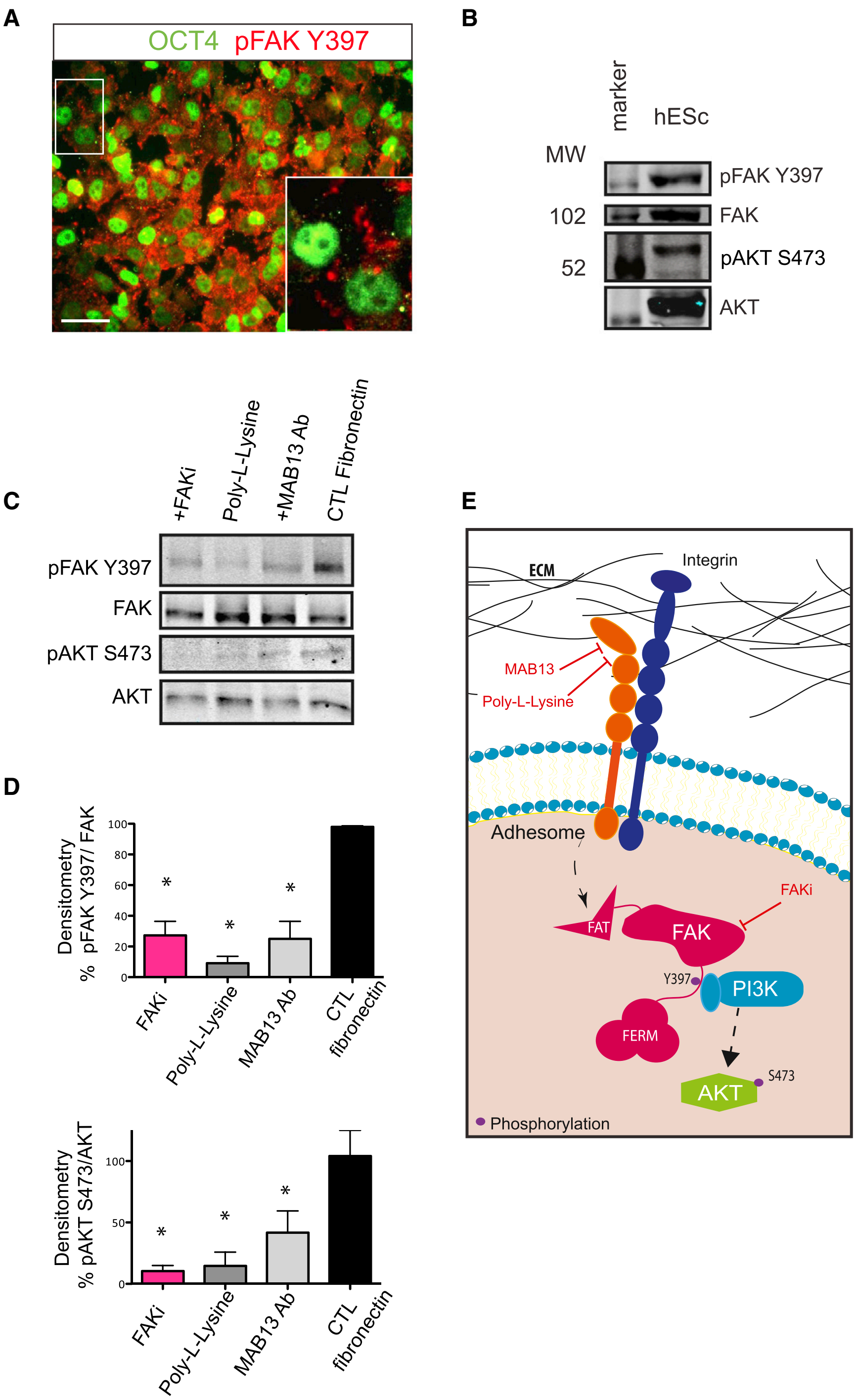

Figure 1. Matrix-Integrin Binding Activates FAK Signaling Upstream of AKT

(A) Immunofluorescence of hESCs cultured on fibronectin for 24 hr and stained with antibodies against OCT4 and pFAKY397. Scale bar, 50 μm.

(B) Immunoblot of pFAKY397, FAK, pAKTS473, and AKT in hESCs stably cultured on fibronectin.

(C) Immunoblot of pFAKY397, FAK, pAKTS473, and AKT in hESCs 1 hr after being plated in the following conditions, on: fibronectin (CTL); fibronectin plus 10 μg/mL of β1-integrin blocking antibody (MAB13); non-integrin activating Poly-L-Lysine substrate or fibronectin plus 2 μM PF562271 (FAKi).

(D) Densitometry of immunoblots for pFAKY397/FAK ratio and pAKTS473/AKT ratio for the conditions in (C). Data represent mean + SEM (n = 3 experiments). *$p < 0.05$ relative to CTL.

(E) Integrin signaling cascade and points of inhibition.

See also Figure S1.

Loss of Total FAK Disrupts hESC Attachment and Leads to Cell Aggregation

Since FAK is a modular protein that functions as a scaffold during adhesome assembly linking integrins to the actin cytoskeleton, we investigated the effect of substantially reducing total FAK on hESCs. Knocking down FAK protein by 70% using small interfering RNA (siFAK) (Figure 2G) induced visible changes in morphology and decreased hESC attachment to fibronectin. Cells formed aggregates similar to those seen after blocking β1-integrin with antibody (Figure 2H), showing that integrin/FAK is required for the transmission of attachment cues in hESCs. Moreover, cell aggregates were also produced in hESCs grown on vitronectin, after blocking its receptor αVβ5-integrin

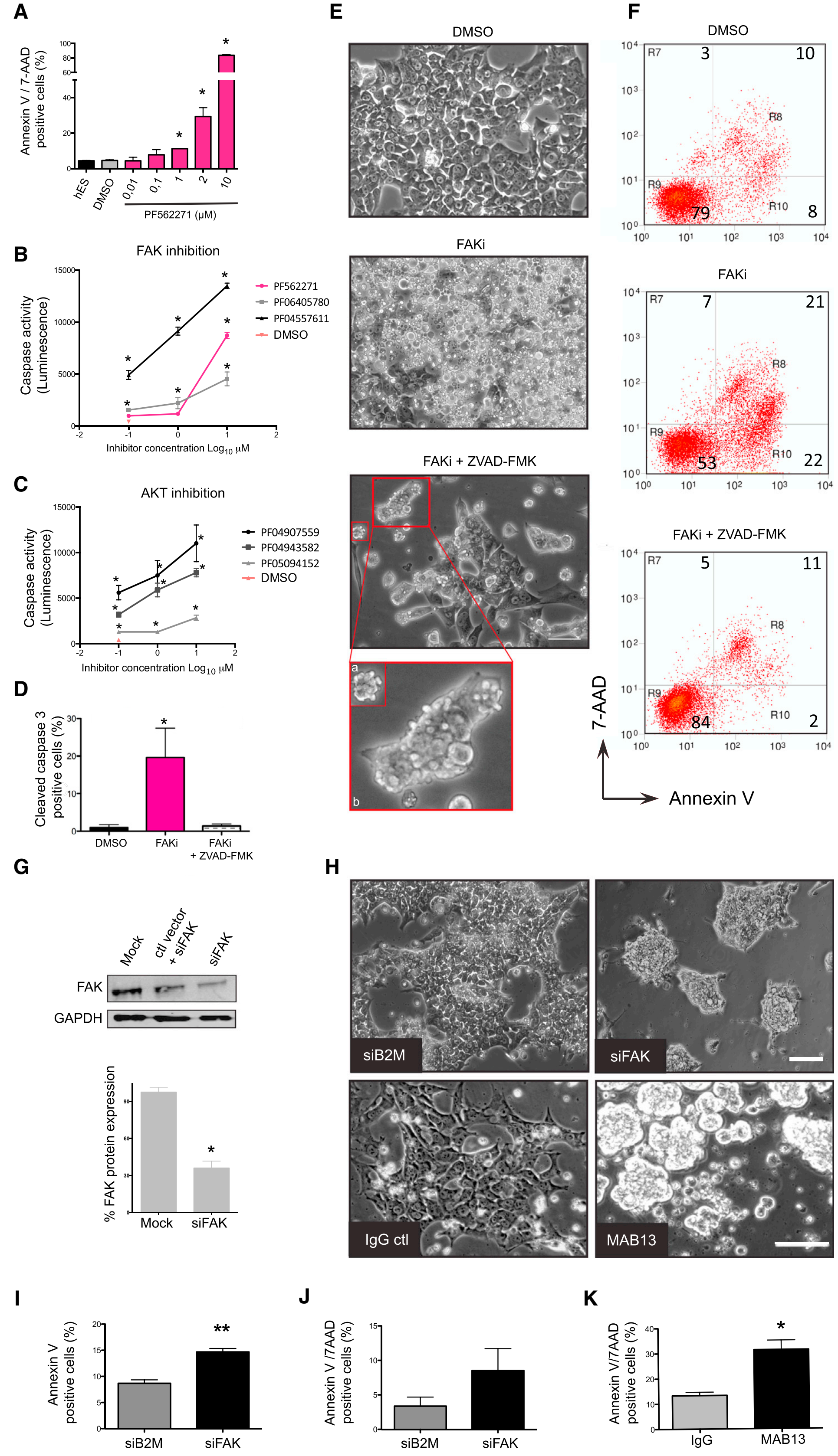

(legend on next page)

with antibody (Figure S2I). Similarly to the effect of MAB13, pFAK activity was downregulated after inhibition of vitronectin-integrin binding (Figure S2J), suggesting that a common downstream FAK signaling may be activated. Furthermore, apoptosis was increased by both MAB13 and siFAK (Figures 2K and 2I), although late apoptotic cells are fewer in the latter (Figure 2J), strongly supporting the role of β1-integrin and FAK in survival of hESCs. In conclusion, loss of β1-integrin activity or FAK produces a shift from matrix-cell adhesion to cell-cell adhesion with lowered survival, highlighting the role of integrin/FAK signaling in the maintenance of pro-survival adhesion cues in hESCs.

FAK Localizes in the Nucleus of hESCs and Regulates MDM2/p53 Levels during Anoikis

The results reported in Figures 1C and 2A link FAK inhibition with the downregulation of AKT and a subsequent apoptotic response. It has been reported that de-adhesion or kinase inhibition promotes nuclear accumulation of FAK (Lim et al., 2012), which exercises kinase-independent functions, including scaffolding MDM2 ubiquitination of p53 as an additional mechanism to support survival (Lim et al., 2008). To test whether FAK participates in this process in hESCs, we first determined its cellular location. Immunofluorescence of hESCs with total FAK antibody revealed a diffuse distribution in the cytoplasm but also in the nucleus, while FAKY397 localized at the cell surface (Figure 3A). Accordingly, FAK was repeatedly found in the nuclear fraction of hESCs (Figure 3B). After 6 hr of integrin blocking or FAK inhibition, total FAK did not accumulate in the nucleus, but was reduced in the cytoplasm and also slightly so in the nucleus (Figures 3C and S3A). Immunofluorescence also showed a widespread loss of FAK after 1 hr from integrin/FAK inhibition (Figure 3D). Moreover, inhibition of integrin/FAK deactivated pMDM2 while upregulating p53 (Figure 3E). The elevation of p53 protein was detectable already after 1 hr of treatments (Figure S3B), and we observed its increase mainly in the nucleus (Figure 3F). Furthermore, p53 upregulation appears linked to the reduced MDM2 ubiquitin ligase activity, since FAK inhibition reduces the poly-ubiquitin chains associated with p53 (Figure 3G). In conclusion, attachment of hESCs to the ECM supports FAK kinase and total protein expression, which keeps MDM2 active and p53 low, ultimately preventing a caspase-dependent anoikis.

hESCs Avoid Anoikis by Exiting Their Undifferentiated State

We have demonstrated that integrin signaling regulates survival of hESCs, but does it play a role in maintaining the undifferentiated stem cell state? First, we found that after 24 hr of FAKi the entire population of apoptotic hESCs retained the pluripotency-associated marker NANOG (Figure 4A). However, when hESCs were cultured in the presence of FAKi for 3 days, a subpopulation of cells that remained attached and escaped anoikis had acquired a differentiated morphology (Figures 4B and S4A). Indeed, the cells were NANOG negative and had elongated nuclei after 2 or 1 μM of FAKi (Figures 4C and S4C). Similar results were obtained on vitronectin and laminin (Figure S4D). Strikingly, FAKi-differentiated cells dramatically downregulated FAK and NANOG proteins while pSMAD2 was slightly increased (Figure 4D). At the gene-expression level, FAKi reduced both *NANOG* and *OCT4* pluripotency-associated genes (Figures 4E and S4B). In parallel, early differentiation genes were upregulated (Figure 4E). Consistent with the role of FAK downstream of integrins, blocking β1-integrin also induced differentiation in a similar fashion (Figure 4F). Integrin-blocked aggregates were comparable with embryoid bodies (EBs) in the induction of differentiation, although EBs showed a greater induction of

Figure 2. Inhibition of FAK Signaling Induces Cell Blebbing and a Caspase-Dependent Anoikis

(A) Quantification of Annexin V/7-AAD positive cells by flow cytometry in hESCs treated for 24 hr with the indicated concentrations of PF562271, DMSO, or untreated. $^*p < 0.05$ relative to DMSO.

(B) Caspase activity in hESCs treated for 5 hr with the indicated concentration of FAK inhibitors. $^*p < 0.05$ relative to DMSO.

(C) Caspase activity in hESCs treated for 5 hr with the indicated concentration of AKT inhibitors. $^*p < 0.05$ relative to DMSO.

(D) Cleaved caspase-3 expression in hESCs treated with DMSO, FAKi only, or with 50 μM ZVAD-FMK for 24 hr. $^*p < 0.05$ relative to DMSO.

(E) Phase images of hESCs treated for 24 hr with DMSO, FAKi only, or with Z-VAD-FMK. Inset shows blebbing in a single cell (a) or groups of cells (b). Scale bar, 100 μm.

(F) Dot plots of Annexin V/7-AAD-positive cells in hESCs treated for 24 hr with DMSO, FAKi only, or with Z-VAD-FMK.

(G) Immunoblot for FAK and GAPDH in hESCs nucleofected with mock control, control GFP vector (ctl vector) plus FAK siRNA (siFAK), or siFAK for 48 hr. Bottom: protein knockdown efficiency. $^*p < 0.05$.

(H) Top: phase images of hESCs after knockdown with β2-microglobulin siRNA (siB2M) control or siFAK for 66 hr. Bottom: hESCs after treatment with 10 μg/mL of IgG isotype or MAB13 antibody for 24 hr. Scale bars, 100 μm (top) and 50 μm (bottom).

(I) Quantification of Annexin V-positive hESCs after knockdown with siB2M or siFAK (66 hr). $^{**}p < 0.03$.

(J) Quantification of Annexin V/7-AAD-positive hESCs after knockdown with siB2M or siFAK (66 hr).

(K) Quantification of Annexin V/7-AAD-positive hESCs treated with IgG or MAB13 antibody (24 hr). $^*p < 0.05$.

Data represent mean + SEM (n = 3 experiments). See also Figure S2.

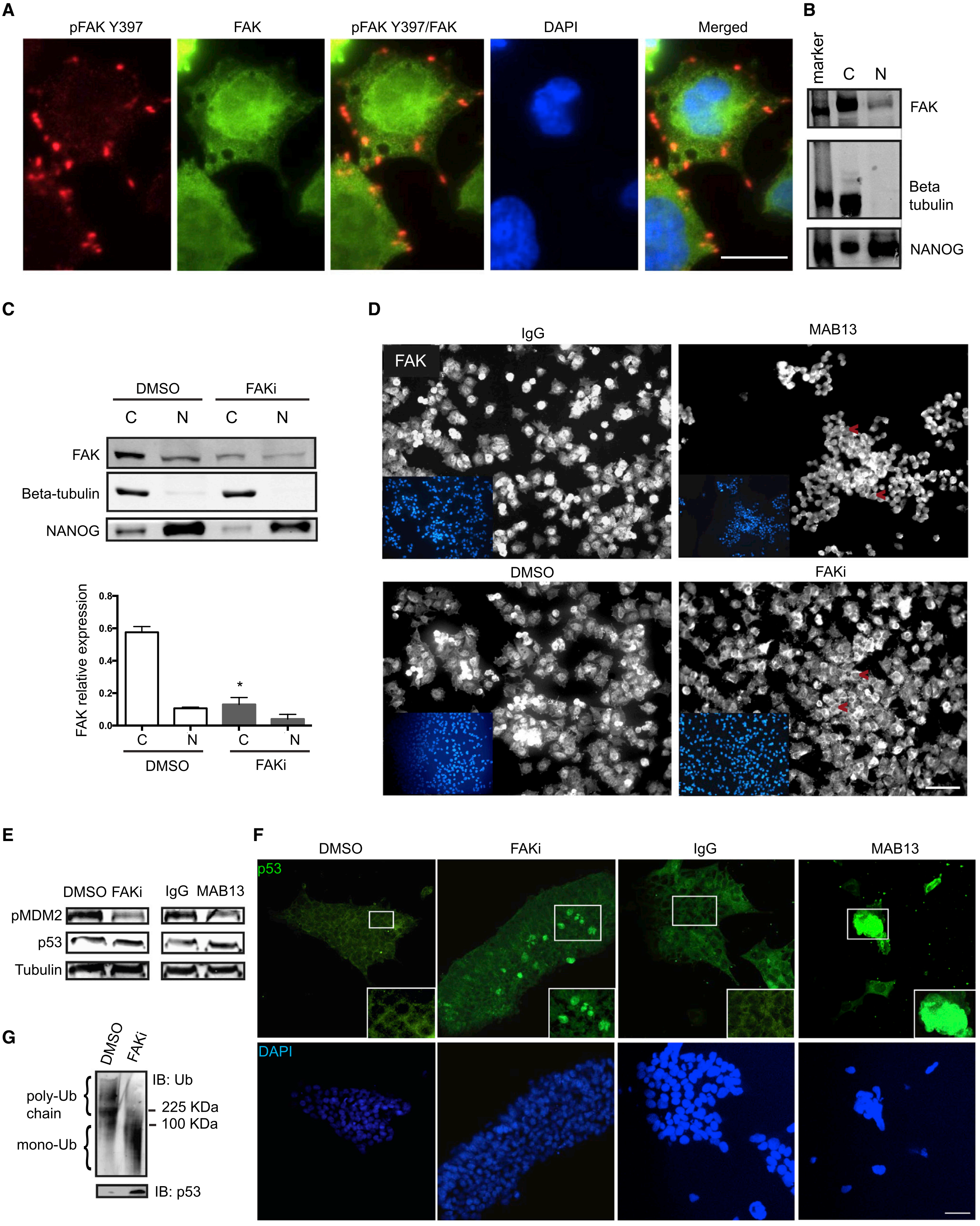

(legend on next page)

differentiation markers at day 3 (Figure 4F), but similar to MAB13 inhibited aggregates at day 5 (Figure S4E). Together, these data show that hESCs undertake one of two routes after inhibition of integrin/FAK signaling: they die through anoikis, remaining undifferentiated, or survive but differentiate, losing characteristic hESCs morphology and markers. Our data highlight a model of hESC regulation by integrin signaling (Figure 4G) with FAK as a major transducer of integrin cues for attachment, survival, and maintenance of stem cell identity.

DISCUSSION

Here we shed light on the events downstream of integrin activation in hESCs and reveal in FAK the mediator of this signaling and a positive regulator of survival, adhesion, and stem cell maintenance. Indeed, hESCs respond to FAK inhibition by exiting the stem cell state through either anoikis or differentiation. We found that integrin activation in hESCs is transduced by FAKY397 to activate AKT and MDM2 and suppress p53 and caspase activation. This FAK-dependent survival pathway is consistent with that reported in adult cell types (Lim et al., 2008), but in the context of hESCs we reveal how integrin signaling supports pluripotency circuits, since AKT and p53 are well known to regulate the balance between self-renewal and differentiation (Singh et al., 2012; Jain et al., 2012). We found no evidence that the loss of FAK signaling in pluripotent cells biases differentiation to a particular lineage, but rather the resultant switch-off of PI3K/AKT and switch-on of p53 can shift hESCs out of pluripotency (Singh et al., 2012; Jain et al., 2012).

Our data confirm and extend previous work reporting FAK and AKT phosphorylation in hESCs (Miyazaki et al., 2012; Rodin et al., 2014; Wrighton et al., 2014). However, a recent paper reported that FAK is activated only upon differentiation (Villa-Diaz et al., 2016), similarly to murine ESCs where stem cell maintenance inversely correlates with integrin activation (Toya et al., 2015). This discrepancy with our and previous reports on hESCs may be explained by our data showing that the integrin signaling players are indeed active in hESCs but are only assembled into obvious focal adhesions upon differentiation. Clearly, hESCs manipulate complex integrin machinery for different purposes in different environments. For example, it was shown that ILK inhibition but not FAK inhibition increased endoderm differentiation in the presence of activin A (Wrighton et al., 2014), whereas we saw no effect of ILK on survival or differentiation when added to hESC media.

We found that hESCs require FAK for maintenance of substrate adhesion, which is consistent with its role in transmitting forces from integrins to the cytoskeleton (Huveneers and Danen, 2009). Double inhibition of FAK kinase and caspase revealed signs of cytoskeletal hypercontraction, similar to the unique and lethal hESC-response to single-cell dissociation (Ohgushi et al., 2010), but also visible in groups of mutually adhering cells. Thus, our results suggest that integrin signaling is an essential yet distinct cue from cell-cell adhesion for suppression of cytoskeleton contraction and apoptosis. However, integrin signaling may crosstalk with cell-cell adhesion. Indeed, we observed formation of cell aggregates after blocking β1-integrin or FAK knockdown.

Finally, we discovered that hESCs possess a nuclear pool of FAK that does not accumulate but reduces after FAK inhibition, unlike in adult cells (Lim et al., 2012). We propose that hESCs utilize non-canonical FAK mechanisms to quickly respond to defective adhesion, which could involve undiscovered roles for nuclear FAK in the context of pluripotent cells.

In conclusion, this study shows that the ECM exerts extensive control over feeder-free hESCs via a FAK-dependent cascade linking integrins to intrinsic regulatory players, supporting survival and maintenance of stem cell

Figure 3. FAK Localizes in the Nucleus of hESCs and Regulates MDM2/p53 Levels during Anoikis

(A) Wide-field fluorescence microscopy images of hESCs co-stained with DAPI and antibodies against pFAKY397 and total FAK. Scale bar, 20 μm.

(B) Immunoblot of FAK in the nuclear and cytoplasmic fraction of hESCs. β-tubulin serves as cytoplasmic (C) marker and NANOG as nuclear (N) enriched marker.

(C) Immunoblot of FAK in the nuclear and cytoplasmic fraction of hESCs treated with DMSO or FAKi for 6 hr. Graph: densitometry. Data represent mean + SEM (n = 3 experiments). $^*p < 0.05$.

(D) Immunofluorescence images of hESCs treated with DMSO, FAKi, IgG, or MAB13 for 1 hr. Cells were co-stained with DAPI and antibodies against FAK. Arrows indicate areas with less FAK staining. Scale bar, 50 μm.

(E) Immunoblot of pMDM2, p53, and β-tubulin in hESCs treated with DMSO, FAKi, IgG, or MAB13 for 6 hr.

(F) Fluorescence microscopy images of hESCs treated with DMSO, FAKi, IgG, or MAB13 for 1 hr. Cells were co-stained with DAPI and antibody against p53. Scale bars, 50 μm.

(G) Immunoblot of ubiquitinated p53 immunoprecipitated from hESCs treated with or without FAKi for 6 hr.

See also Figure S3.

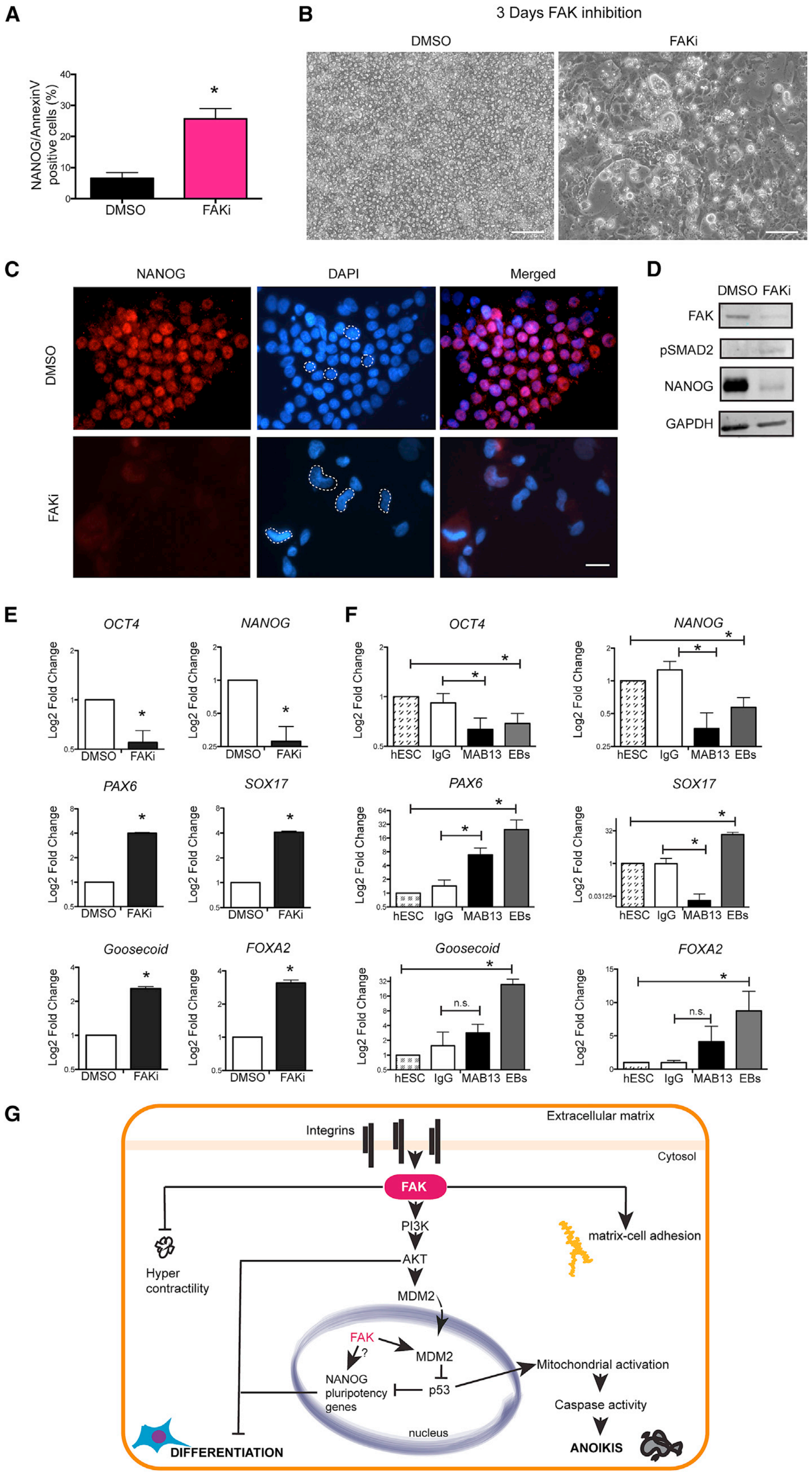

(legend on next page)

identity. This study advances our understanding of the underlying integrin signaling controlling hESCs, of particular importance for their culture on ECM for both basic and translational research.

EXPERIMENTAL PROCEDURES

Cell Culture

hESCs HUES1 (Harvard University), MAN5 (North West Embryonic Stem Cell Center), Shef-1 (University of Sheffield), and hiPSCs Sendai J (Neusentis) were cultured in defined mTSER1 (Life Technologies) medium on 50 μg/mL fibronectin as previously described (Baxter et al., 2009). Cells were passaged with TryPLE express (Gibco) at a ratio of 1:3. HUES1 was used as lead line throughout the study, MAN5 was used when specified, and Shef1 and SendaiJ to confirm compound screening. For selected experiments, hESCs were plated on 5 μg/mL of vitronectin-N (Life Technologies) or laminin 521 (Biolamina).

Integrin Blocking

hESCs were dissociated and seeded at a density of 0.7×10^4 cells/cm^2 in hESC media supplemented with 10 μg/mL MAB13 anti β1-integrin antibody (M. Humphries, University of Manchester) or rat immunoglobulin G (IgG) isotype (Sigma) and plated on fibronectin. For cells cultured on vitronectin, αVβ5-integrin was inhibited with 10 μg/ml MAB1916z blocking antibody (Millipore) or mouse IgG isotype (Sigma). For antibody-free and EB controls, hESCs were seeded at the same density on either fibronectin-coated or non-adherent tissue-culture plates, respectively.

Compound Screening

Cells were prepared 24 hr before the assay by passaging in presence of Y-27632 (10 μM) (Sigma-Aldrich) to a fibronectin-coated 96-well plate (Cell-BIND, Corning) at a density of 2×10^4 cells/well. Cells were treated in triplicate with FAK or AKT inhibitors, at the concentrations indicated in the figures, added to mTESR1 medium, and incubated for 5 hr at 37°C. The ApoToxGlo Triplex assay (Promega) was used according to the manufacturer's instructions. Signals were analyzed with a SpectraMax M5 reader (Molecular Devices).

siRNA Knockdown

hESCs were treated with 10 μM Y27632 (Sigma-Aldrich) for 1 hr and nucleofected with Amaxa Nucleofector following the manufacturer's instructions (Lonza), with target-specific FAK siRNA (sc-29310, Santa Cruz Biotechnology) at a final concentration of 200 nM, β-2-microglobulin siRNA control (Life Technologies), or pmaxGFP control vector (Lonza). Medium was changed the following day and experimental analysis performed 66 hr later.

Statistics

Data were analyzed with Student's *t*-test using GraphPad Prism. p values of <0.05 were considered significant.

SUPPLEMENTAL INFORMATION

Supplemental Information includes Supplemental Experimental Procedures, four figures, and one table and can be found with this article online at http://dx.doi.org/10.1016/j.stemcr.2016.07.006.

AUTHOR CONTRIBUTIONS

L.V. designed and performed the experiments; M.B. performed integrin analysis; B.I. performed the Src and integrin αVβ5 experiments; P.W. supervised the compound screening; S.J.K. designed and supervised the study; L.V. and S.J.K. co-wrote the manuscript.

ACKNOWLEDGMENTS

The authors thank Prof. Martin Humphries for the gift of MAB13 and 12g10 antibodies, Dr. Andrew Gilmore for discussion, and Dr. Christoph Ballestrem for microscopy. This work was supported by an MRC CASE studentship (G1000399) in collaboration with Neusentis to L.V., a Turkish Ministry of National Education (MoNE 1416) studentship to B.I., and NW Science fund grant (N0003382) and MRC grant (G0801057) to S.J.K.

Received: January 26, 2016
Revised: July 8, 2016
Accepted: July 8, 2016
Published: August 9, 2016

Figure 4. hESCs Avoid Anoikis by Exiting Their Undifferentiated State
(A) Quantification of Annexin V/NANOG double-positive hESCs treated for 24 hr with DMSO or FAKi. *p < 0.05.
(B) Phase images of hESCs treated with DMSO or FAKi for 3 days. Scale bar, 100 μm.
(C) Immunofluorescence images of hESCs treated with DMSO or FAKi for 3 days. Cells were co-stained with DAPI and antibody against NANOG. Scale bar, 50 μm.
(D) Immunoblot of FAK, pSMAD2, and NANOG in hESCs treated with DMSO or FAKi for 3 days.
(E) Gene expression showing fold change for pluripotency-associated markers *NANOG* and *OCT4* and early differentiation markers *PAX6*, *SOX17*, *Goosecoid*, and *FOXA2* in hESCs treated with DMSO or FAKi for 3 days. *p < 0.05 relative to DMSO.
(F) Gene expression fold change for pluripotency-associated and early differentiation markers [as in (E)] in hESCs treated with 10 μg/ml of MAB13 or IgG control for 3 days. *p < 0.05, MAB13 relative to IgG and EBs relative to hESCs; n.s., not significant.
(G) Proposed model for FAK signaling in hESCs: ECM-integrin binding activates FAK, which induces PI3K upstream of AKT/MDM2 survival cascade leading to suppression of p53. In absence of FAK activity, the concomitant switch-off of AKT and elevation of p53 induces a caspase-dependent anoikis or downregulation of hESCs core genes and differentiation.
Data represent mean + SEM (n = 3 experiments). See also Figure S4.

REFERENCES

Baxter, M.A., Camarasa, M.V., Bates, N., Small, F., Murray, P., Edgar, D., and Kimber, S.J. (2009). Analysis of the distinct functions of growth factors and tissue culture substrates necessary for the long-term self-renewal of human embryonic stem cell lines. Stem Cell Res. *3*, 28–38.

Braam, S.R., Zeinstra, L., Litjens, S., Ward-van Oostwaard, D., van den Brink, S., van Laake, L., Lebrin, F., Kats, P., Hochstenbach, R., Passier, R., et al. (2008). Recombinant vitronectin is a functionally defined substrate that supports human embryonic stem cell self-renewal via alphavbeta5 integrin. Stem Cells *26*, 2257–2265.

Coleman, M.L., Sahai, E.A., Yeo, M., Bosch, M., Dewar, A., and Olson, M.F. (2001). Membrane blebbing during apoptosis results from caspase-mediated activation of ROCK I. Nat. Cell Biol. *3*, 339–345.

Frame, M.C., Patel, H., Serrels, B., Lietha, D., and Eck, M.J. (2010). The FERM domain: organizing the structure and function of FAK. Nat. Rev. Mol. Cell Biol. *11*, 802–814.

Frisch, S.M., and Francis, H. (1994). Disruption of epithelial cell-matrix interactions induces apoptosis. J. Cell Biol. *124*, 619–626.

Giancotti, F.G., and Ruoslahti, E. (1999). Integrin signaling. Science *285*, 1028–1032.

Gilmore, A.P., Metcalfe, A.D., Romer, L.H., and Streuli, C.H. (2000). Integrin-mediated survival signals regulate the apoptotic function of Bax through its conformation and subcellular localization. J. Cell Biol. *149*, 431–446.

Huveneers, S., and Danen, E.H. (2009). Adhesion signaling - crosstalk between integrins, Src and Rho. J. Cell Sci. *122*, 1059–1069.

Jain, A.K., Allton, K., Iacovino, M., Mahen, E., Milczarek, R.J., Zwaka, T.P., Kyba, M., and Barton, M.C. (2012). p53 regulates cell cycle and microRNAs to promote differentiation of human embryonic stem cells. PLoS Biol. *10*, e1001268.

Lim, S.T., Chen, X.L., Lim, Y., Hanson, D.A., Vo, T.T., Howerton, K., Larocque, N., Fisher, S.J., Schlaepfer, D.D., and Ilic, D. (2008). Nuclear FAK promotes cell proliferation and survival through FERM-enhanced p53 degradation. Mol. Cell *29*, 9–22.

Lim, S.T., Miller, N.L., Chen, X.L., Tancioni, I., Walsh, C.T., Lawson, C., Uryu, S., Weis, S.M., Cheresh, D.A., and Schlaepfer, D.D. (2012). Nuclear-localized focal adhesion kinase regulates inflammatory VCAM-1 expression. J. Cell Biol. *197*, 907–919.

Miyazaki, T., Futaki, S., Suemori, H., Taniguchi, Y., Yamada, M., Kawasaki, M., Hayashi, M., Kumagai, H., Nakatsuji, N., Sekiguchi, K., et al. (2012). Laminin E8 fragments support efficient adhesion and expansion of dissociated human pluripotent stem cells. Nat. Commun. *3*, 1236.

Nichols, J., and Smith, A. (2009). Naive and primed pluripotent states. Cell Stem Cell *4*, 487–492.

Ohgushi, M., Matsumura, M., Eiraku, M., Murakami, K., Aramaki, T., Nishiyama, A., Muguruma, K., Nakano, T., Suga, H., Ueno, M., et al. (2010). Molecular pathway and cell state responsible for dissociation-induced apoptosis in human pluripotent stem cells. Cell Stem Cell *7*, 225–239.

Parsons, J.T. (2003). Focal adhesion kinase: the first ten years. J. Cell Sci. *116*, 1409–1416.

Rodin, S., Antonsson, L., Niaudet, C., Simonson, O.E., Salmela, E., Hansson, E.M., Domogatskaya, A., Xiao, Z., Damdimopoulou, P., Sheikhi, M., et al. (2014). Clonal culturing of human embryonic stem cells on laminin-521/E-cadherin matrix in defined and xeno-free environment. Nat. Commun. *5*, 3195.

Singh, A.M., Reynolds, D., Cliff, T., Ohtsuka, S., Mattheyses, A.L., Sun, Y., Menendez, L., Kulik, M., and Dalton, S. (2012). Signaling network crosstalk in human pluripotent cells: a Smad2/3-regulated switch that controls the balance between self-renewal and differentiation. Cell Stem Cell *10*, 312–326.

Soteriou, D., Iskender, B., Byron, A., Humphries, J.D., Borg-Bartolo, S., Haddock, M.C., Baxter, M.A., Knight, D., Humphries, M.J., and Kimber, S.J. (2013). Comparative proteomic analysis of supportive and unsupportive extracellular matrix substrates for human embryonic stem cell maintenance. J. Biol. Chem. *288*, 18716–18731.

Toya, S.P., Wary, K.K., Mittal, M., Li, F., Toth, P.T., Park, C., Rehman, J., and Malik, A.B. (2015). Integrin alpha6beta1 expressed in ESCs Instructs the differentiation to endothelial cells. Stem Cells *33*, 1719–1729.

Villa-Diaz, L.G., Kim, J.K., Laperle, A., Palecek, S.P., and Krebsbach, P.H. (2016). Inhibition of FAK signaling by integrin alpha6beta1 supports human pluripotent stem cell self-renewal. Stem Cells *34*, 1753–1764.

Wrighton, P.J., Klim, J.R., Hernandez, B.A., Koonce, C.H., Kamp, T.J., and Kiessling, L.L. (2014). Signals from the surface modulate differentiation of human pluripotent stem cells through glycosaminoglycans and integrins. Proc. Natl. Acad. Sci. USA *111*, 18126–18131.

Xia, H., Nho, R.S., Kahm, J., Kleidon, J., and Henke, C.A. (2004). Focal adhesion kinase is upstream of phosphatidylinositol 3-kinase/Akt in regulating fibroblast survival in response to contraction of type I collagen matrices via a beta 1 integrin viability signaling pathway. J. Biol. Chem. *279*, 33024–33034.

Stem Cell Reports, Volume 7

Supplemental Information

Integrin-Associated Focal Adhesion Kinase Protects Human Embryonic Stem Cells from Apoptosis, Detachment, and Differentiation

Loriana Vitillo, Melissa Baxter, Banu Iskender, Paul Whiting, and Susan J. Kimber

SUPPLEMENTAL FIGURES

FIGURE S1

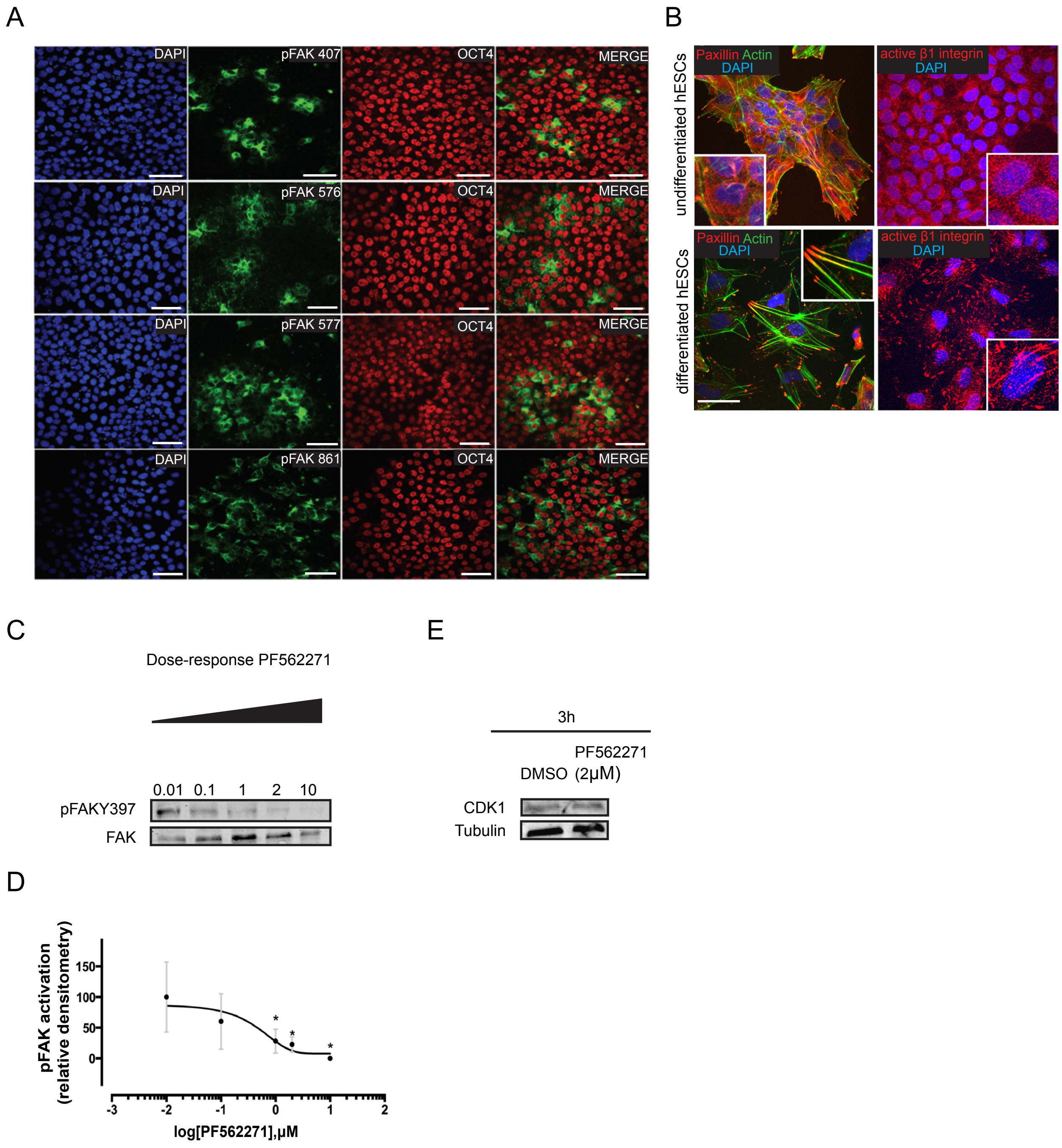

Figure S1. Matrix-Integrin Binding Activates FAK Signaling Upstream of AKT (Related to Figure 1)

(A) Immunofluorescence images of hESCs cultured on fibronectin and stained with antibodies against OCT4 and pFAKY407, pFAKY576, pFAKY577 or pFAKY861. Scale bars 50 μm
(B) Immunofluorescence image of undifferentiated and differentiated hESCs cultured on fibronectin and stained with antibodies against Paxillin, Actin and active β1-integrin. For differentiation, cells were grown in hESCs media (Baxter et al., 2009) without NT4 and AA for 7 days. Scale bar 50 μm
(C) Immunoblot of pFAKY397 and FAK after increasing concentrations of PF562271 inhibitor added for 1 h.
(D) Dose-response curve of pFAKY397 relative to PF562271 concentration added for 1 h. Average of 3 independent experiments. *indicates $p<0.05$.
(E) Immunoblot of CDK1 and β-tubulin after 3 hr of 2 μM PF562271.

Data represented as mean +SEM

FIGURE S2

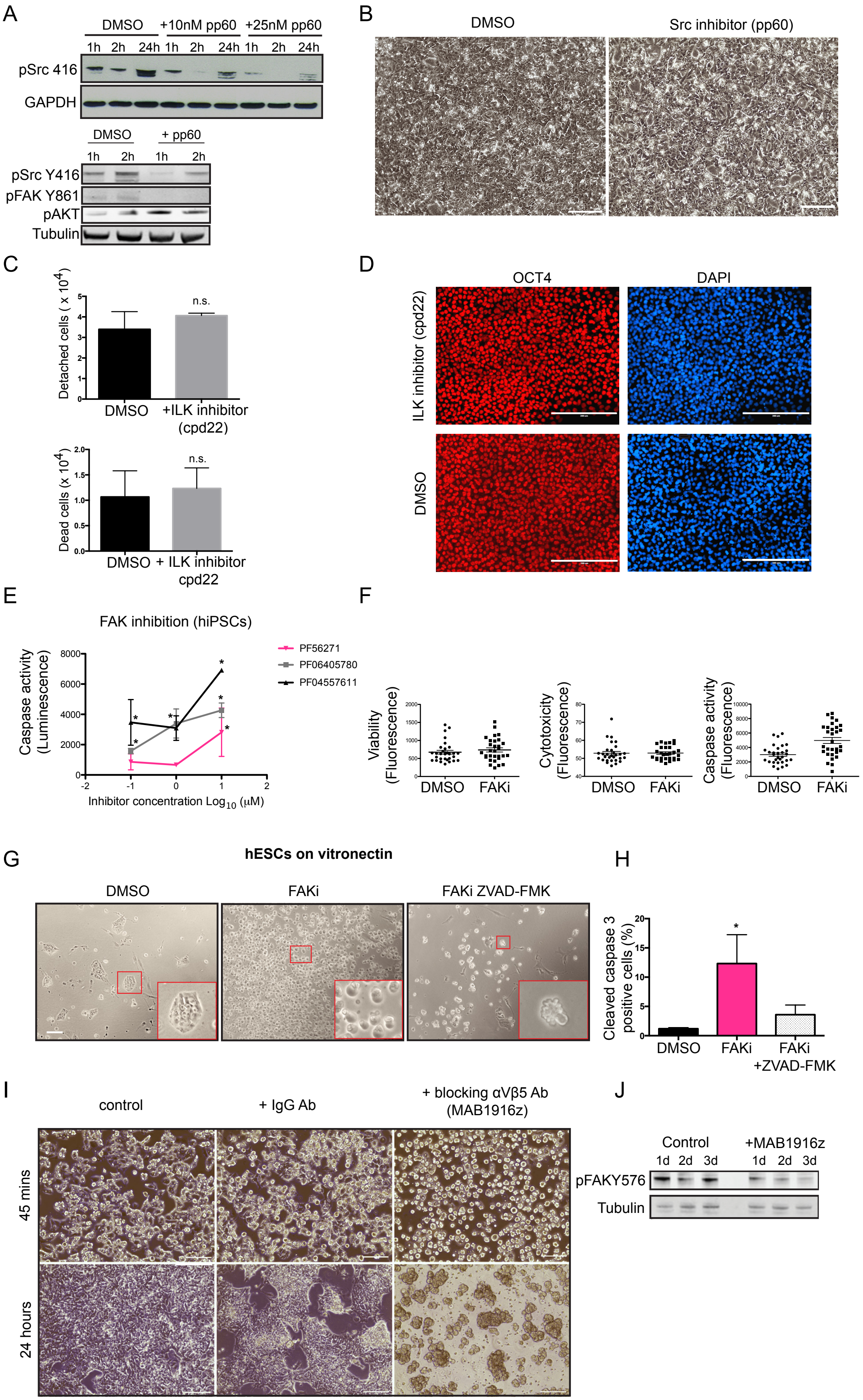
A
DMSO
+10nM pp60
+25nM pp60
1h 2h 24h 1h 2h 24h 1h 2h 24h
pSrc 416
GAPDH
DMSO
+ pp60
1h 2h 1h 2h
pSrc Y416
pFAK Y861
pAKT
Tubulin
B
DMSO
Src inhibitor (pp60)
C
Detached cells (x 10^4)
n.s.
DMSO
+ILK inhibitor (cpd22)
Dead cells (x 10^4)
n.s.
DMSO
+ ILK inhibitor cpd22
D
OCT4
DAPI
ILK inhibitor (cpd22)
DMSO
E
FAK inhibition (hiPSCs)
Caspase activity (Luminescence)
PF56271
PF06405780
PF04557611
Inhibitor concentration Log10 (μM)
F
Viability (Fluorescence)
Cytotoxicity (Fluorescence)
Caspase activity (Fluorescence)
DMSO
FAKi
G
hESCs on vitronectin
DMSO
FAKi
FAKi ZVAD-FMK
H
Cleaved caspase 3 positive cells (%)
DMSO
FAKi
FAKi +ZVAD-FMK
I
control
+ IgG Ab
+ blocking αVβ5 Ab (MAB1916z)
45 mins
24 hours
J
Control
+MAB1916z
1d 2d 3d 1d 2d 3d
pFAKY576
Tubulin

Figure S2. Inhibition of FAK Signaling Induces Cell Blebbing and a Caspase-Dependent Anoikis (Related to Figure 2)

(A) Top: Immunoblot of pSrc416 and GAPDH in hESCs treated with DMSO or Src inhibitor pp60 at 10nM or 25nM for 1h, 2h or 24hr. Bottom: Immunoblot of pSrc416, pFAKY861, pAKT and β-tubulin in hESCs treated with DMSO or pp60 (25nM) for 1 and 2 hr.
(B) Phase images of hESCs plated on fibronectin for 24 hr in presence of Src inhibitor pp60 (25 nM) or DMSO control. Scale bars 100 μm
(C) Quantification of cell detachment (cell in suspension) and survival (dead cells stained positive with trypan blue) in hESCs treated for 24 hr with 0.4 μM of ILK inhibitor cpd22. (n=3 independent experiments).
(D) Immunofluorescence images of hESCs treated with DMSO or 0.4 μM cpd22 for 3 days and stained with antibody against OCT4.
(E) Caspase activity in hiPSCs treated for 5 hr with the indicated concentrations of FAK inhibitors. *indicates $p<0,05$ (n=3 independent experiments).
(F) Scatter plots of viability, cytotoxicity and caspase activity measured in parallel in hiPSCs treated with DMSO or 2 μM of PF562271 for 5 hr. Each dot represents a well of a 96-well plate.
(G) Phase images of hESCs cultured on vitronectin substrate and treated for 24 hr with DMSO, FAKi only or with Z-VAD-FMK. Inserts from left to right show: hESCs colony, cells undergoing anoikis and cell blebbing. Scale bar 50 μm
(H) Cleaved-caspase 3 expression in hESCs cultured on vitronectin and treated with DMSO, FAKi only or with Z-VAD-FMK for 24 hr. *indicates $p<0.05$ relative to DMSO (n=3 independent experiments).
(I) Phase images of hESCs replated on vitronectin for 45 mins or 24 hr in presence of 10 μg/ml of MAB1916z or mouse IgG control versus untreated control. Scale bars 100 μm.
(J) Immunoblot of pFAKY576 or β-tubulin in hESCs cultured on vitronectin from 1 to 3 days (3d) with 10 μg/ml of IgG control or MAB1916z.

Data represented as mean +SEM

FIGURE S3

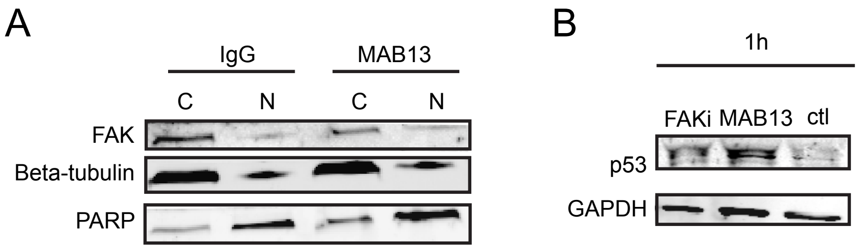

Figure S3. FAK Localizes in the Nucleus of hESCs and Regulates MDM2/p53 Levels during Anoikis (Related to Figure 3)

(A) Immunoblot of FAK in the nuclear and cytoplasmic fraction of hESCs treated with IgG control or MAB13 for 6 hr. β-tubulin serves as cytoplasmic marker (C) and PARP as nuclear marker (N).
(B) Immunoblot of p53 and GAPDH in control hESCs versus cells treated with FAKi or MAB13 for 1 hr.

FIGURE S4

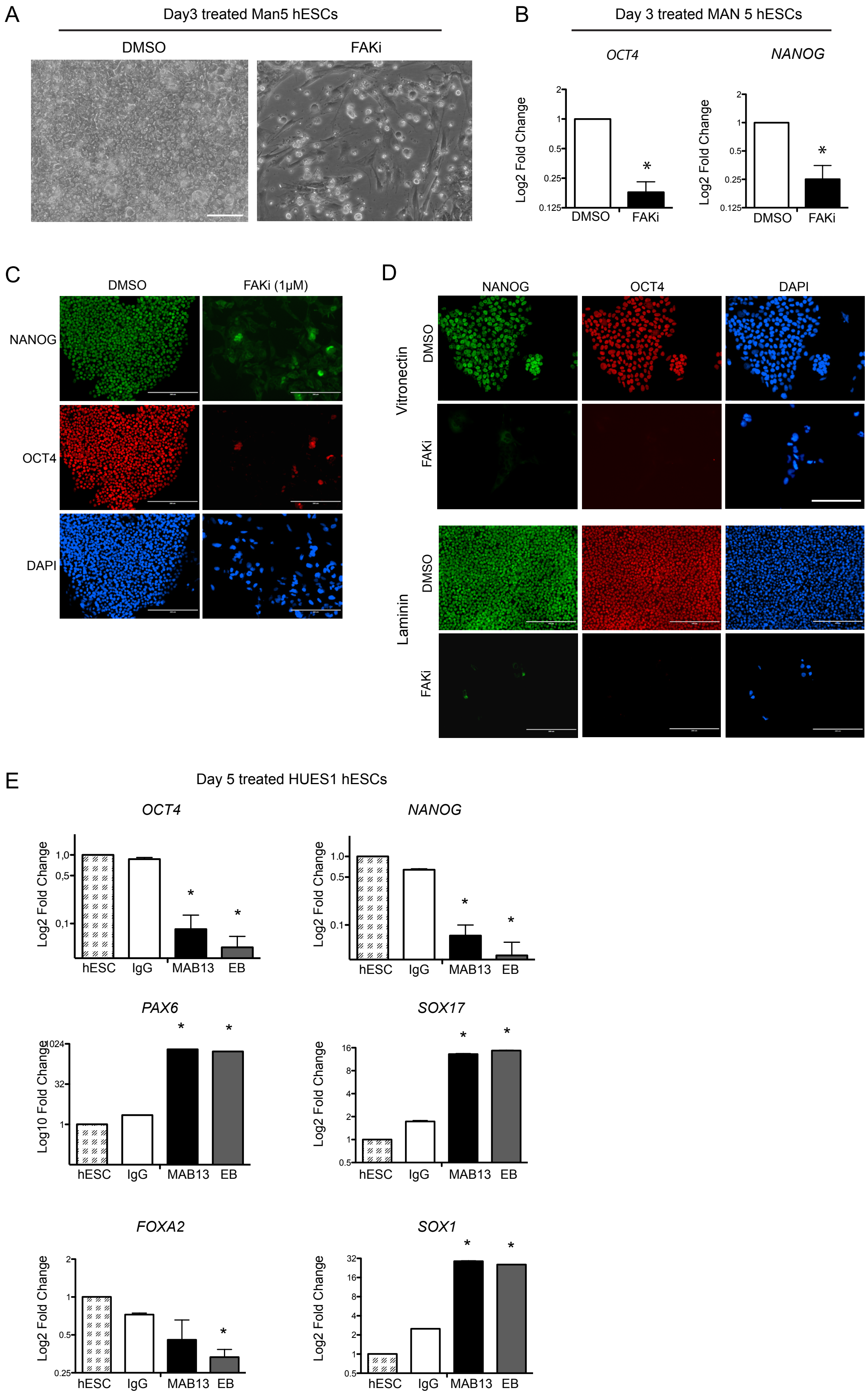
A
Day3 treated Man5 hESCs
DMSO
FAKi
B
Day 3 treated MAN 5 hESCs
OCT4
NANOG
Log2 Fold Change
DMSO
FAKi
C
DMSO
FAKi (1μM)
NANOG
OCT4
DAPI
D
NANOG
OCT4
DAPI
Vitronectin
Laminin
DMSO
FAKi
E
Day 5 treated HUES1 hESCs
OCT4
NANOG
PAX6
SOX17
FOXA2
SOX1
Log2 Fold Change
Log10 Fold Change
hESC
IgG
MAB13
EB

Figure S4. hESCs Avoid Anoikis by Exiting their Undifferentiated State (Related to Figure 4)

(A) Phase images of hESCs MAN5 treated with DMSO or 2 μM of PF562271 (FAKi) for 3 days. Scale bar 100 μm.
(B) Gene expression fold-change of pluripotency markers *NANOG* and *OCT4* in hESC MAN5 treated with DMSO or FAKi for 3 days. * indicates p<0,05 (n=3 independent experiments).
(C) Immunofluorescence images of hESCs cultured on fibronectin and treated with DMSO or 1 μM PF562271 for 3 days. Cells were costained with DAPI and antibodies against NANOG and OCT4. Scale bars 200 μm.
(D) Immunofluorescence images of hESCs cultured on vitronectin or laminin 521 and treated with DMSO or 2 μM PF562271 for 3 days. Cells were costained with DAPI and antibodies against NANOG and OCT4. Scale bars 200 μm.
(E) Gene expression fold-change of pluripotency markers *NANOG*, *OCT4* and differentiation markers *PAX6*, *SOX17*, *FOXA2* and *SOX1* in hESC HUES1 treated with DMSO, FAKi, 10μg of MAB13 or rat IgG for 5 days. * indicates p<0,05. MAB13 relative to IgG, EB relative to hESCs (n=3 independent experiments)

Data represented as mean +SEM

SUPPLEMENTAL EXPERIMENTAL PROCEDURES

Apoptosis assay and Flow Cytometry:
Apoptosis measurements were performed using the Annexin V-PE and 7-AAD kit (BD Pharmigen) and acquired on a Lab Quanta SC flow cytometer (Beckman Coulter). For Annexin V/NANOG double staining, samples were pre-stained with Annexin V for 15 mins and washed 3 times with PBS 1X prior to fixation. For intracellular staining, cells were fixed in 4% paraformaldehyde at room temperature and permeabilized in 70% MeOH at 4°C. Samples were probed with Nanog-PE or AlexaFluor 488 Isotype control (both from BD Pharmigen) for 30 mins and analysed. Data were analysed using Summit 4.3 (Beckman Coulter).

Immunofluorescence:
hESCs were fixed in 4% paraformaldehyde, blocked with 10% serum matching the species of the secondary antibody and permeabilised with 0.1% TritonX-100. Cells were incubated with primary antibodies dilutions containing 1% serum overnight at 4°C. Cells were washed and incubated with secondary antibodies conjugated with Alexa fluorophores (Invitrogen) and nuclei stained with DAPI (Sigma). Images were taken with Olympus 1X71 inverted microscope (Olympus) with a Q-imaging Retiga SRV camera and processed using QCapture Pro (QImaging) and ImageJ 64.

Widefield restoration microscopy with Delta Vision:
hESCs were grown and fixed on glass-bottom culture dishes (35.0/10mm Grainer Bio-one) coated with fibronectin. Images were acquired on a Delta Vision Olympus 1X71 microscope (Applied Precision) restoration microscope using a 100x/140 Uplan S Apo objective and the Sedat Quad filter set. The images were collected using a Coolsnap HP (Photometrics) camera with a Z optical spacing of 0.2 μm.

Antibodies and reagents:
Immunofluorescence antibodies were used against: Paxillin (BD Bioscience), F-Actin (Fluorescein phalloidin, Invitrogen), activated β1-integrin 12g10 (gift from prof. M.Humphries, Wellcome Matrix Centre, The University of Manchester), NANOG (R&D Systems); OCT4 (BD Biosciences); FAK clone 4.47 (Millipore); pFAKY397, pFAKY407, pFAKY576, pFAKY577 and pFAKY861 (Invitrogen); p53 (ab131442, Abcam), Cleaved-caspase3 (Asp175, Cell Signalling).
Western blotting antibodies were against: FAK, pFAKY397, pFAK576, pSrc416, pAKTSer473, p53, pMDM2, NANOG (all from Cell Signalling); pFAK861 (Invitrogen); CDK1 (R&D); GAPDH 71.1 (Sigma Aldrich); AKT (Invitrogen); pSMAD2 (Millipore); Multiubiquitin Chain MoAb (clone FK2, Cayman chemical); β-tubulin (Li-COR). Secondary antibodies: IRDye 680LT Goat anti-Mouse IgG; IRDye 800CW Goat anti-Rabbit IgG (Li-COR), IRDye 680LT Donkey anti-Goat IgG.
Small molecules: FAK inhibitors PF-562271 (Roberts et al., 2008), PF-06405780 (Bunnage et al., 2011), PF-04557611 (Lovering et al., 2012); AKT inhibitors PF-05094152 (Heerding et al., 2008), PF-04907559 (Heerding et al., 2007) and PF-04943582 (Zhu et al., 2007) were all provided by Pfizer. ILK inhibitor cpd22 from Calbiochem. Src inhibitor pp60 (521-533) from Tocris. Caspase inhibitor Z-VAD-FMK from Abcam.

Protein extraction and immunoblotting:
Adherent cells were lysed on ice in RIPA buffer (Sigma) with added protease and phosphatase inhibitors (ROCHE). Protein extracts were quantified using the BCA protein detection kit (Thermo Scientific). Equal protein amounts were separated on 12% poly-acrylamide gel and transferred to a nitrocellulose membrane (Wathman). Membranes were blocked in Odyssey buffer (Li-COR), incubated overnight at 4°C with primary antibody, washed and incubated with IRDye conjugated secondary antibodies for 2 hr at room temperature. Membranes were scanned with the Odyssey detection system (Li-COR).

Subcellular fractionation:
hESCs pellets were lysed in NER-PER extraction reagents (Thermo Scientific). Cell pellet were resuspended in 200 μl of CERI buffer plus protease and phosphatase inhibitors (ROCHE) for 10 mins on ice. CERII buffer was added to the solution, vortexed for 15 mins, incubated 2 mins on ice, vortexed 5 sec and spun at 500 g for 7 mins. Cytoplasmic proteins were collected from the supernatant. The remaining nuclear pellet was washed twice in PBS, spun at 500 g for 7 min and incubated in 50 μl of NER buffer for 40 mins on ice, vortexed every 10 mins. Nuclear proteins were collected in the supernatant after a 16,000 g centrifugation for 10 mins at 4°C.

Immunoprecipitation:
hESCs pellet corresponding to 5 x 10^6 cells was used for each immunoprecipitation condition following the p53 immunocapture kit (ab154470, Abcam). For each immunoprecipitation, 10 μl of p53 beads slurry were used in a 1.5 ml tube incubated under rotation for 16 hr at 4°C.

Real-time PCR:
Total RNA was isolated using the RNeasy Mini Kit (Qiagen) and converted to cDNA with the M-MLV reverse transcriptase (Promega). For qPCR mastermixes, power SYBR Green Master Mix (Applied Biosystems) was used and plates were run using the CFX96 Real-Time System (Bio-Rad). mRNA levels were normalized to GAPDH expression and relative expression calculated as fold-change from the untreated control.

Table S1. Primer set used in this study, Related to Figure 4

GENE	FORWARD	REVERSE
OCT4	AGACCATCTGCCGCTTTGAG	GCAAGGGCCGCAGCTT
NANOG	GGCTCTGTTTTGCTATATCCCCTAA	CATTACGATGCAGCAAATACAAGA
GAPDH	ATGGGGAAGGTGAAGGTCG	TAAAAGCAGCCCTGGTGACC
PAX6	CTGGCTAGCGAAAAGCAACAG	CCCGTTCAACATCCTTAGTTTATCA
SOX17	AGAGATTTGTTTCCCATAGTTGGATT	TGTTTTGGGACACATTCAAAGCT
SOX1	GCGGTAACAACTACAAAAAACTTGTAA	GCGGAGCTCGTCGCATT
FOXA2	TTCAGGCCCGGCTAACTCT	AGTCTCGACCCCCACTTGCT
Goosecoid	GATGCTGCCCTACATGAACGT	GACAGTGCAGCTGGTTGAGAAG

SUPPLEMENTAL REFERENCES

Baxter, M.A., Camarasa, M.V., Bates, N., Small, F., Murray, P., Edgar, D., and Kimber, S.J. (2009). Analysis of the distinct functions of growth factors and tissue culture substrates necessary for the long-term self-renewal of human embryonic stem cell lines. Stem Cell Res 3, 28-38.

Bunnage, M.E., Cook, A.S., Cui, J.J., Dack, K.N., Deal, J.G., Gu, D., He, M., Johnson, P.S., Johnson, T.W., Le, P.T.Q., et al. (2011). Preparation of heterocyclic derivatives such as 2-aminopyridine and 3-aminopyridazine derivatives as anaplastic lymphoma kinase (ALK) inhibitors for the treatment of diseases. PCT Int. Appl. WO 2011138751 A2 20111110.

Heerding, D.A., Clark, T.J., Leber, J.D., Safonov, I.G. (2007). 1H-inidazo[4,5-c]pyridin-2-yl derivatives as inhibitors of AKT activity and their therapeutic uses. PCT Int. Appl. WO 2007058850 A2 20070524.

Heerding, D.A., Rhodes, N., Leber, J.D., Clark, T.J., Keenan, R.M., Lafrance, L.V., Li, M., Safonov, I.G.,Takata, D.T., Venslavsky, J.W., et al. (2008). Identification of 4-(2-(4-aminso-1,2,5-oxadiazol-3-yl)-1-ethyl-7-{[(3S)-3-piperidinylmethyl]oxy}-1H- imidazo[4,5-c]pyridin-4-yl)-2-methyl-3-butyn-2-ol (GSK690693), a novel inhibitor of AKT kinase. J Med Chem 51, 5663-5679.

Lovering, F., McDonald, J., Whitlock, G.A., Glossop, P.A., Phillips, C., Bent, A., Sabnis, Y., Ryan, M., Fitz, L.,Lee, J., et al. (2012). Identification of type-II inhibitors using kinase structures. Chem Biol Drug Des 80, 657-664.

Roberts, W.G., Ung, E., Whalen, P., Cooper, B., Hulford, C., Autry, C., Richter, D., Emerson, E., Lin, J., Kath, J., et al. (2008). Antitumor activity and pharmacology of a selective focal adhesion kinase inhibitor, PF-562,271. Cancer Res 68, 1935-1944.

Zhu, G.D., Gandhi, V.B., Gong, J., Thomas, S., Woods, K.W., Song, X., Li, T., Diebold, R.B., Luo, Y., Liu, X., etal. (2007). Syntheses of potent, selective, and orally bioavailable indazole-pyridine series of protein kinase B/Akt inhibitors with reduced hypotension. J Med Chem 50, 2990-3003.
